# Decoding Cancer-Associated Mutations in DNA Polymerase η through Atomistic Simulations

**DOI:** 10.1021/acs.jctc.6c00345

**Published:** 2026-05-21

**Authors:** Alessia Visigalli, Paolo Carloni, Marco De Vivo

**Affiliations:** † Laboratory of Molecular Modeling & Drug Discovery, 121451 Istituto Italiano di Tecnologia, Via Enrico Melen 83, 16142 Genoa, Italy; ‡ Computational Biomedicine, Institute of Neuroscience and Medicine INM-9, Forschungszentrum Jülich GmbH, 52428 Jülich, Germany; § Department of Physics, RWTH Aachen University, 52056 Aachen, Germany; ∥ JARA Institute: Molecular Neuroscience and Imaging, Institute of Neuroscience and Medicine INM-11, Forschungszentrum Jülich GmbH, 09042 Jülich, Germany

## Abstract

DNA polymerases (Pols) are essential enzymes for DNA replication within the cell. However, DNA lesions, such as cyclobutane pyrimidine dimers (CPDs) induced by ultraviolet (UV) radiation, can impair Pols’s function and DNA replication. Therefore, the presence of CPDs can, for example, lead to xeroderma pigmentosum variant (XP-V), a rare genetic disorder characterized by an increased risk of skin cancer. Nonetheless, in these situations, specific translesion synthesis (TLS) Pols, such as human DNA polymerase η (Polη), can overcome such lesions, enabling DNA polymerization. That is, Polη prevents the pathological risks associated with CPD-caused DNA replication stalling. Here, we analyzed how a selected set of 8 Polη mutations perturbs its structure, DNA binding, and substrate translocation, thereby altering Polη function, preventing it from bypassing CPDs, thus making them XP-V pathogenic mutations. Leveraging recent structural and clinical data on these pathogenic Polη variants, we elucidated the mechanistic basis for their impairment of Polη′s ability to bypass damage. We employed molecular dynamics simulations to examine their effects on Polη in pre- and post-translocation states. Although these residues vary in location and chemical nature, we found that all contributed to reducing DNA anchoring to Polη. In this way, we could identify a unified mechanistic framework for decoding how XP-V pathogenic mutations compromise Polη function by destabilizing the Polη–DNA complex.

## Graphical abstract

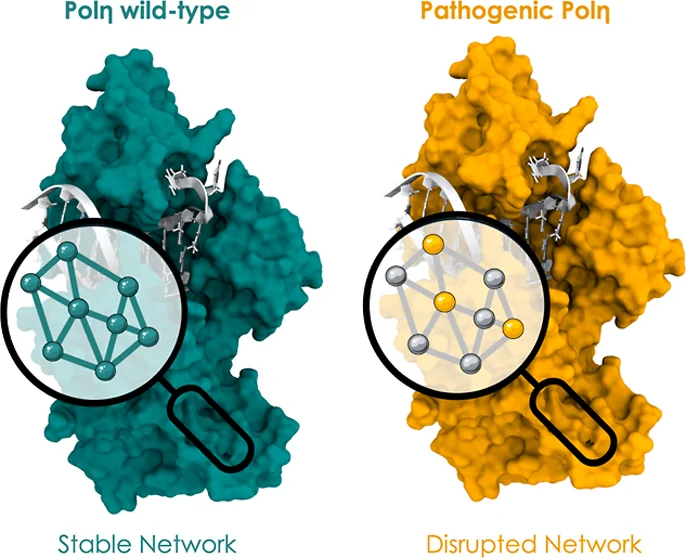

## Introduction

Preserving genomic stability under genotoxic stress conditions is essential for cell survival across the three domains of life.^1,2^ When replicative DNA polymerases (Pols) encounter DNA lesions, the high-fidelity DNA synthesis is blocked.^3^ Thus, cells rely on translesion synthesis (TLS), a damage-tolerance pathway mediated by specialized low-fidelity DNA polymerases, primarily members of the Y-family.^4^ These enzymes are uniquely adapted to accommodate and bypass bulky DNA lesions within their enlarged active sites.^4,5^ Among them, human DNA polymerase η (Polη) plays a crucial role in bypassing UV-induced cyclobutane pyrimidine dimers (CPDs), one of the most prevalent forms of DNA damage arising from solar radiation.^6,7^

Polη synthesizes DNA by sequential incorporation of deoxynucleotides (dNTP) at the 3′ terminus of the primer strand during a multistep catalytic cycle, involving four states (I–IV in Figure 1A).^8–10^ The binary Polη·DNA_*n*_ complex recruits the dNTP and the metal ions from the bulk, forming the Polη·DNA_*n*_·dNTP·2Mg^2+^ complex (I).^10^ The presence of the two metal ions assists the catalytic step, in which the nucleophilic attack of the 3′–OH group of the growing strand leads to phosphodiester bond formation (II).^10^ Simultaneously, the phosphodiester bond between the phosphates α and β of the dNTP is cleaved, allowing the release of the pyrophosphate group (PPi) and the metal ions.^11–15^ In the next pre-translocation state (III), the Mg^2+^ ions are absent,^16,17^ while second-shell sodium ions are transiently present.^18^ At this point, DNA translocation can occur.^18^ Then, the enzyme is ready for a new cycle (I–IV), with the growing DNA strand now one nucleotide longer (IV). A wealth of structural and biochemical studies on Polη have shown that it adopts a characteristic right-handed architecture composed of the palm, finger, thumb, and little-finger (LF) subdomains.^4,8,19^ Together, these four subdomains surround the DNA substrate and define the active-site environment­(Figure 1B-C).^4,8,19–22^ The palm subdomain hosts the conserved DED catalytic triad (Asp15, Asp115, and Glu116), responsible for coordinating the divalent metal ions required for catalysis (Figure 1A).^10,23–25^ The fingers subdomain stabilizes the dNTP, while the thumb and LF subdomains, enriched in positively charged residues, are critical for binding the DNA duplex and promoting DNA translocation.^10,18^

**Figure 1:**
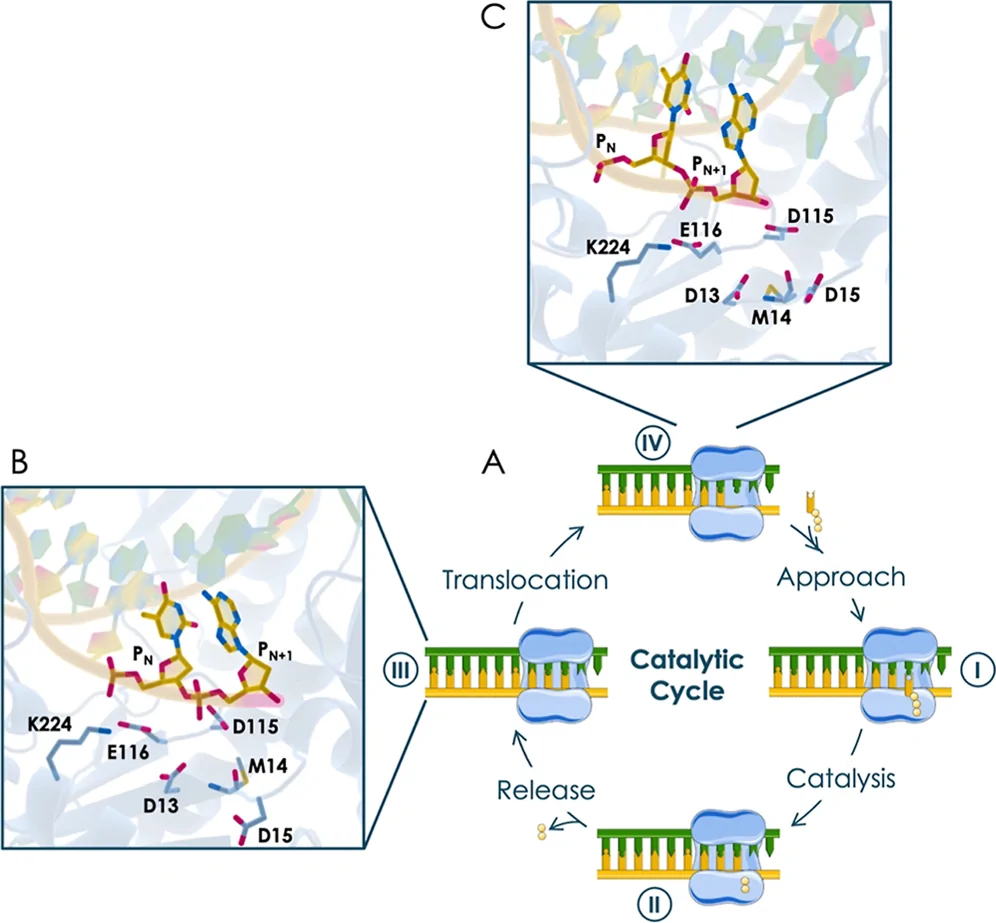
Catalytic cycle of DNA polymerase η and structural models of the states investigated in this study. (A) Schematic representation of the catalytic cycle of DNA polymerase η. (I) Pre-catalytic state, in which binding of the incoming nucleotide leads to formation of the ternary complex Polη·DNA_*n*_·dNTP. (II) Post-catalytic state following phosphodiester bond formation, with the newly incorporated nucleotide and the pyrophosphate (PPi) located in the active site. (III) Pre-translocation state, corresponding to the binary complex Polη·DNA_*n*_ after release of the Mg^2+^ ions and PPi. (IV) Post-translocation state, in which the DNA has translocated and the complex Polη·DNA_*n*+1_ is poised for a new catalytic cycle. The template strand is shown in green, the primer strand in yellow, and the polymerase in light blue. (B) Atomistic MD structure of the pre-translocation state (state III) used in this work, derived from the corresponding X-ray structure (PDB ID: 4ECX) and previously characterized in Visigalli et al.^18^ Key catalytic residues are shown as sticks and colored by atom type. Protein and nucleic acids are represented as cartoons. (C) Atomistic MD structure of the post-translocation state (PDB ID: 4ED8; state IV) used in this study, represented using the same color scheme as in panel B.

In humans, the loss or impairment of Polη activity may lead to xeroderma pigmentosum variant (XP-V), a rare autosomal recessive disorder characterized by extreme UV sensitivity and a markedly increased predisposition to skin cancer.^2,22,26–29^ Unlike other XP subtypes, which arise from defects in nucleotide excision repair (NER), XP-V results exclusively from mutations in the POLH gene, encoding Polη.^22^ To date, more than 40 XP-V–associated POLH mutations have been identified across nearly 50 XP-V patients (Table S1).^22,27–29^ These variants can be broadly divided into two main classes based on their impact on protein function. The majority of them (∼75%) consist of truncations, deletions, or insertions that introduce deleterious frameshifts, abolishing enzyme function. By contrast, only a limited number of missense mutations have been reported (∼25%). Notably, a subset of these missense variants appears to target the catalytic core of Polη. In particular, eight mutations (Arg93Pro, Arg111His, Ala117Pro, Thr122Pro, Gly263Val, Val266Asp, Gly295Arg, and Arg361Ser) cluster within this region (Table S1), suggesting potential effects on catalytic activity or substrate interactions. Although these are clinically pathogenic, their impact on enzyme structure, DNA binding, and catalytic function remains largely unknown^30,31^ This lack of mechanistic understanding is striking, given the wealth of structural and computational data available for the wild-type (WT) Polη enzyme, including detailed descriptions of DNA binding, nucleotide incorporation, and translocation dynamics.^8,10,11,18,24,32^ Notably, inspection of available Polη structures^22^ further reveals that the XP-V–associated missense mutations are positioned near regions essential for metal-ion coordination, electrostatic stabilization of the DNA substrate, and interdomain communication, suggesting that these mutations may all converge onto common functional disruptions (Figure 2, left).^8,22,26,29^

**Figure 2:**
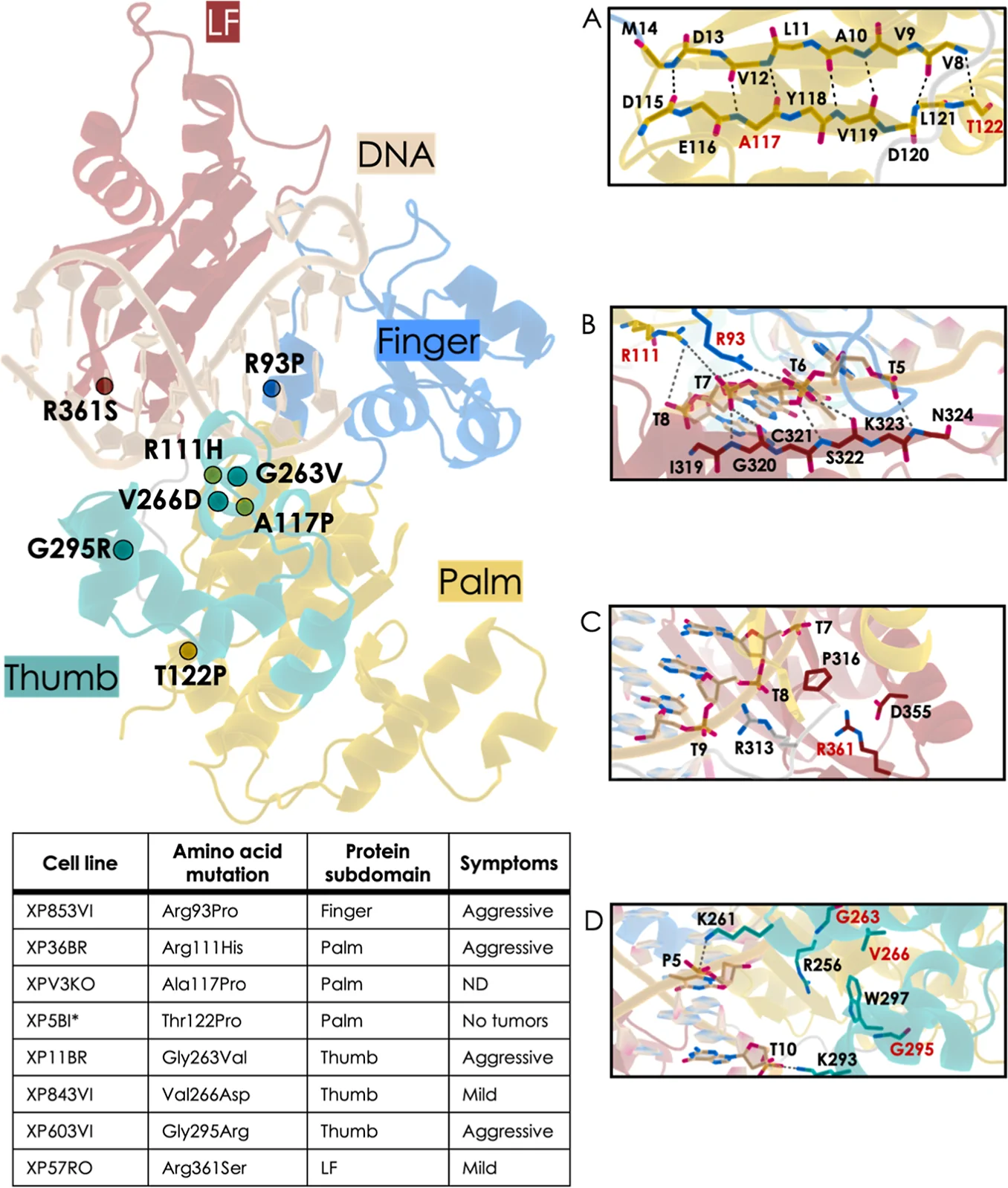
Structural localization of XP-V–associated missense mutations in human DNA polymerase η (Polη). (Left) Cartoon representation of the catalytic core of wild-type (WT) Polη in the pre-translocation state (state III; PDB ID: 4ECX). The protein is colored according to its four subdomains: palm (gold), fingers (blue), thumb (teal), and little finger (firebrick). The eight XP-V–associated missense mutations investigated in this study are shown as spheres, colored by subdomain, and listed in the table below. (Right) Close-up views of the local structural environments of the mutated residues, grouped by functional regions and colored by subdomain. (A) The β-hairpin of the palm subdomain, where Ala117 and Thr122 are structurally coupled to the catalytic triad. (B) The Finger–Palm interface, where Arg93 and Arg111 interact with the template strand and contribute to stabilization of the molecular splint. (C) The C-terminal region, where Arg361 stabilizes the helix containing Asp355. (D) The hydrophobic patch of the Thumb subdomain, comprising Gly263, Val266, and Gly295. Clinical information on the patients and the phenotypic severity associated with each XP-V mutation is summarized in the table shown in the lower left panel.

Here, we used atomistic molecular dynamics (MD) simulations to investigate how these eight missense XP-V–associated mutations perturb the structure and dynamics of Polη during translocation.^33–35^ We focused on the pre- and post-translocation states (states III and IV), which lack metal ions at the catalytic site but exhibit critical protein–DNA interactions.^16–18^ By comparing the dynamics of all eight missense mutations with the WT enzyme in complex with a defined primer-template duplex, we identified convergent structural and interaction-based signatures that suggest impaired DNA binding and translocation in these mutated systems.

## Results

To elucidate how XP-V–associated mutations affect the structural dynamics of Polη during DNA synthesis, we performed classical molecular dynamics (MD) simulations in both the pre-translocation (state III) and post-translocation (state IV) states. By comparing these two mechanistically distinct conformations, we aimed to determine whether the mutations perturb the enzyme differently, before and after the translocation step, thereby impacting DNA binding, subdomain coordination, and overall structural stability. Here, we simulated eight XP-V–associated missense mutations (Arg93Pro, Arg111His, Ala117Pro, Thr122Pro, Gly263Val, Val266Asp, Gly295Arg, and Arg361Ser) in complex with the DNA duplex (Figure 2). While the overall Polη–DNA complex remained rather stable, we noted that such mutations induced global deviations in specific subdomains, which propagated to the protein–DNA interface (Figure 3).

**Figure 3:**
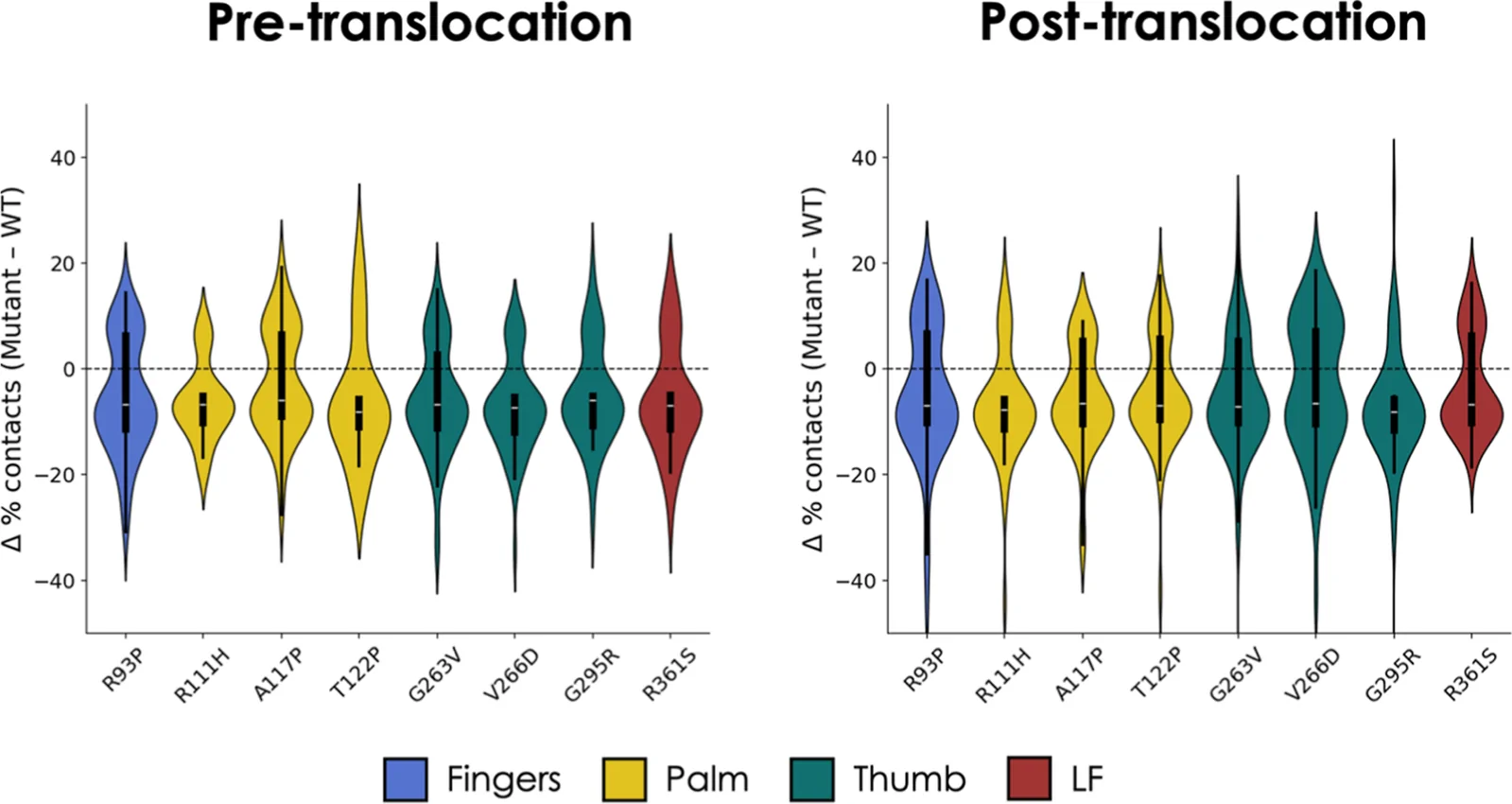
Distribution of mutation-induced changes in protein–DNA contacts across XP-V variants. Violin plots depict the per-residue change in contact frequency (Δ%) between WT and mutant Polη for each XP-V–associated missense mutation. The left panel shows results in state III, while the right panel shows results in state IV. The width of each violin represents the probability density of Δ% values, and the internal boxplots show the interquartile range (Q1–Q3) and median. The dashed horizontal line at Δ% = 0 indicates no change relative to the wild type. Mutations are colored according to the subdomain in which the affected residue resides (Palm: gold; Finger: blue; Thumb: teal; Little-Finger: firebrick), highlighting that structural perturbations occur both at the active site and at distal DNA-binding interfaces.

### Local Mutations Destabilize the Catalytic Geometry and DNA Coordination in the Pre-translocation Complex (State III)

Here, we collected multiple MD simulations for each single-point mutation in the state III (PDB ID: 4ECX; ∼1 μs, three replicas). This state represents the system after nucleotide incorporation, in the absence of metal ions and ligands. Overall, the palm subdomain appeared stable (Figures S1–S8), with the DNA substrate remaining anchored to the enzyme throughout the simulation. Despite global stability, all simulated mutations affected the active-site geometry, Polη–DNA interactions, and subdomain flexibility. The Ala117Pro mutation introduced a kink that perturbed the active-site geometry. This local perturbation altered ion coordination. Although Mg^2+^ ions were not explicitly simulated, the catalytic triad in the WT system retained the ability to capture and coordinate sodium ions from the bulk. In particular, the two active site residues Asp115 and Glu116 became closer (4.0 ± 0.2 Å in Ala117Pro; 6 ± 1 Å in WT). Concomitantly, the distance between Glu116 and Lys224 increased (6 ± 2 Å in Ala117Pro; 3.5 ± 0.3 Å in WT). This local rearrangement impaired the proper coordination of Na^+^ ions in the A-site (Figure S9). Additionally, the Asp15 side-chain flipping observed in the WT system was completely absent in the Ala117Pro variant (Figure S10). This lack of large motions of the Asp15 side chain impaired the coordination of Na^+^ ions in the B-site (Figure S9). Thus, Na^+^ coordination was significantly weakened, as reflected by a reduction in interactions between the catalytic residues and the cations (Figures S9 and S11). Thr122Pro perturbed the same β-strand, albeit differently: the Glu116-Lys224 distance remained close to WT values (3.4 ± 0.4 Å) and the Asp115-Glu116 distance (6.8 ± 0.3 Å) was compatible with the WT geometry, suggesting partial preservation of sodium ion coordination (Figures S9 and S11). These differences between the two variants suggested that Thr122Pro had a stronger ability to coordinate the A-site cations than Ala117Pro in state III. Intriguingly, our simulations also revealed a previously unobserved interaction between Glu116 and Arg256 (Figure S12). This interaction, absent in all WT replicas and only rarely observed in Ala117Pro, appeared to correlate with the absence of Na^+^ cations in the A-site. Regarding the ability to coordinate ions at the B-site, Asp15 remained able to flip between in and out (Figure S13).

At the Polη–DNA interface, Arg93Pro reduced template strand stability, despite partially recruiting sodium ions from the bulk, revealing a compensatory electrostatic stabilization (Figure S14). The radial distribution function (RDF) showed an increased density of sodium ions near Pro93 compared to the WT, indicating an electrostatic stabilization mechanism unique to the mutant (Figure S15). Notably, the active site H-bond network remained largely intact, and the catalytic triad continued to coordinate Na^+^ ions, suggesting that Arg93Pro is less detrimental for catalysis than the Ala117Pro and Thr122Pro mutants. The Arg111His variant alternated between conformations interacting with the DNA template or with Ser96, as observed in the WT simulations (Figure S16). Intriguingly, His111 predominantly interacted with the phosphate of T8 (3.7 ± 0.2 Å), whereas Arg111 in the WT interacted with T7 (6.6 ± 0.5 Å), potentially affecting the efficiency of DNA translocation. His111 formed π-stacking interactions with Pro316 (4.3 ± 0.6 Å) as found in the WT, adopting a side-chain orientation almost perpendicular to it. This instability is exacerbated by Arg313 in the linker region, which is flexible and solvent-exposed. His111 could form a salt bridge with Arg313 (4.7 ± 0.8 Å), preventing productive interactions with the DNA template and destabilizing the Polη–DNA complex. Similar to the Arg93Pro variant, the Arg111His mutation also appeared to partially compensate for the loss of positive charge by recruiting Na^+^ ions from solution (Figure S15). In the Arg361Ser variant, replacing the bulky, positively charged arginine with a smaller serine disrupted the local interaction network and induced a side-chain flip of Gln395 (Figure S17A), leading to a rearrangement of the major LF helix (Figure S19), which is critical for coordinating the template junction (Figure S18). This helix is crucial for proper enzyme function, as it lies at the interface between the LF and finger subdomains. In this region, multiple protein–DNA interactions occurred, many involving positively charged residues that bind to the DNA substrate and are essential for DNA translocation. In response to this movement, the DNA is slightly displaced and rotated in tandem with the LF, ensuring that the molecular splint remains parallel to the template and maintains DNA contacts (Figure S18). As a result, the thumb subdomain lost its interaction with the DNA. Here, our simulations revealed an increase in sodium ion density near the mutation, compensating for the loss of Arg361 positive charge (Figure S17B). This means that, although the active site and bound sodium ions remained integral, the primer is misaligned, potentially affecting both the catalytic step and DNA translocation.

In the thumb subdomain, Gly263Val introduced steric hindrance that propagated through the H-bond network (Figure S19), detaching the thumb from the DNA (Figure S17C).^36,37^ Interestingly, these local perturbations also propagated to Trp267, a residue likely involved in recruiting the incoming nucleotide through π-stacking interaction with the nucleobase. Indeed, our simulations highlighted a complete detachment of the thumb subdomain from the DNA substrate (Figure S17C). Notably, the Polη–DNA interactions involving the thumb subdomain with the primer strand were markedly different from those of the WT system. Val266Asp and Gly295Arg destabilized the helix spanning residues 260–271, which interacted directly with the template strand (Figure 2). Similar to Gly263Val, these mutations increased thumb mobility relative to the WT and perturbed the positioning of Trp267. Taken together, these observations suggested that local perturbations induced by each single-point mutation could propagate across the system, leading to fewer DNA interactions with the enzyme. For this reason, the DNA duplex became slacker, more mobile, even in the presence of mutations not located at the protein–DNA interface. The instability of the DNA substrate found in state III could prevent proper DNA translocation.

### Mutations Lead to Persistent DNA Misalignment and Subdomain Instability Despite Partial Catalytic Recovery in the Post-Translocation Complex (State IV)

To evaluate the impact of the eight pathogenical mutations in the systems after the translocation process, we collected microsecond-long state IV simulations (PDB ID: 4ED8; ∼1 μs, three replicas). Here, the DNA is shifted by one base pair, compared to state III. Thus, the active site in state IV is empty and ready for the next binding event. Despite most mutants appearing more stable than in state III, our simulation revealed local and global rearrangements in state IV (Figures S21–S28). We observed that active-site–proximal mutants, Ala117Pro and Thr122Pro, retained the geometric distortion of the active site observed in state III. However, the catalytic β-strand geometry was partially preserved from the WT system. Indeed, Ala117Pro showed Glu116–Lys224 at 3.4 ± 0.3 Å and Asp115–Glu116 at 6.7 ± 0.7 Å, values that closely match the WT values (3.5 ± 0.4 Å and 6.6 ± 0.7 Å). These results suggested that the Ala117Pro variant could better coordinate sodium ions at the A-site in state IV. Regarding the B-site, we observed that Met14 frequently flips its carbonyl oxygen, moving from Asp115 (4.1 ± 0.4 Å) to 6.2 ± 0.7 Å. By contrast, this suggested a minor ability of the Ala117Pro variant to coordinate sodium ions in the B-site in state IV. Similarly, the Thr122Pro variant preserved the ability to properly coordinate ions in the A-site, as observed in state III. Indeed, the probability of sodium ions in the active site was lower in the Ala117Pro variant than in the Thr122Pro mutation, which seems unaffected (Figure S29). These findings suggested that, in state IV, the catalytic site geometry is more closely preserved relative to the WT than in state III in the presence of Ala117Pro and Thr122Pro mutations.

Arg93Pro preserved active-site interactions observed in the WT state IV. Notably, the density of sodium ions near the mutation site was weaker than that observed in state III (Figure S15B).^37^ These findings suggested a stronger impact of this mutation in state IV, but it is also possible that the Arg93Pro substitution affects the DNA translocation step or exerts a greater influence in the presence of DNA lesions. Arg111His showed greater instability in the thumb and LF subdomains (Figure S24). By contrast, the active site interactions remained intact, as did the coordination of the sodium ions. As observed for the Arg93Pro variant, the compensatory effect of sodium ions was lower than in state III (Figure S15). In agreement with observationsin state III, Arg361Ser destabilized the LF subdomain, increasing DNA misalignment (Figure S30). Indeed, sodium density near the mutation site increased, suggesting that the compensatory mechanism was preserved from state III (Figure S17B). Additionally, the primer density in the active site was consistent with state III, indicating that the same DNA misalignment caused by the mutation was present.

Finally, thumb mutations (Gly263Val, Val266Asp, Gly295Arg) persistently weakened the DNA interactions, producing perturbations comparable to those observed in state III. Specifically, the interactions involving the thumb subdomain resembled those observed in state III, particularly for residues interacting with the primer strand, such as Gly259, Lys261, and Leu262, as well as Arg351 and Arg93 interacting with the template strand (Figure S31).

### Distinct XP-V–Variants Converge on a Common Loss of DNA Engagement via Distributed Structural Perturbations

Despite the diversity in location and chemical nature of the eight investigated XP-V–associated mutations, our simulations revealed a set of strikingly common structural features in all systems. That is, our MD simulations across all variants and both states (III and IV) showed a loss of DNA grip at the protein–DNA interface. This was mostly due to the propagation of local rearrangements at the protein–DNA interface (Figure 3). This spread occurred regardless of whether mutations were located within the catalytic site, protein–DNA interface, or distal structural elements, indicating that perturbations propagated throughout the whole protein–DNA complex. Notably, ten protein residues (Asp13, Phe17, Phe18, Trp42, Ala49, Ser113, Asp115, Lys224, Ser322, and Leu381) lost their ability to interact with the DNA in state III (Figure 3A). The two active-site residues, Asp13 and Asp115, increased their distance from the last added nucleotide (−15% and −19%, respectively, relative to the WT), and with Phe17 and Phe18 (−9% and −12%). These results suggested that mutations in different regions of the protein misaligned the primer strand with respect to the active site, possibly compromising the catalytic step. Furthermore, the lower interaction of the positive residue Lys224 with P8 (−13%) could compromise the proper translocation of the primer strand. Nevertheless, our simulations revealed the rearrangement of seven residues (Glu116, Gly259, Gly260, Arg351, Asp375, Arg377, and Arg382), indicating that they could now interact with different DNA bases, in line with observations in the WT system (Figure 3B). Notably, most of them are positively charged residues. Arg351 had a stable interaction with T7 in the WT (4.2 ± 0.2 Å). This stable interaction was often present in Ala117Pro (−28%), Gly263Val (−34%), Arg111His (−8%), Arg93Pro (−7%), and Thr122Pro (−13%). By contrast, the percentage increased in Arg361Ser (+17%), Gly295Arg (+19%), and Val266Asp (+5%). Arg377 was interacting with P5 (3.9 ± 0.3 Å) and P4 (4.8 ± 0.3 Å) in the WT system, often flipping its side chain. In our simulations of the eight mutants, Arg377 showed a higher percentage of P5 interactions, while interactions with P4 decreased. Arg382 remained in close contact with P3 in both WT states, an interaction that decreases by almost 9% in all eight mutants, in favor of an increase in interaction with T9 (+4%). This local rearrangement of positively charged residues in the LF subdomain suggested a loss of grip on DNA and a deficiency in DNA translocation. Finally, Arg256 was the only residue found to increase the interaction percentage (+6%) in state III (Figure 3C).

Notably, our simulations revealed fewer residues (Arg93, Lys261, Asn294, Leu315, Glu347, and Arg383) with decreased contact percentages in state IV (Figure 4A). Arg93 stably interacted with T6 in the WT system (4.4 ± 0.3 Å), while the eight mutants led to a decrease of 23% with T6. Again, this WT stable interaction could compromise DNA translocation. Interestingly, Arg383 interacted alternately with both P3 (5.9 ± 0.6 Å) and P4 (4.9 ± 0.3 Å), in both WT states. Here, while the interactions with P3 remained close to the WT values (−0.4%), the interactions with P4 decreased by almost 10% with respect to the WT state IV. Losing the ability to act like a screen wiper for Arg383 could be associated with an impediment in DNA translocation. Regarding a rearrangement in protein–DNA contacts, 14 residues (Gly46, Arg61, Lys86, Arg111, Ala112, Ile225, Arg256, Ser257, Leu258, Gly259, Gly260, Pro316, Arg351, and Arg377) changed their interaction network in the presence of XP-V–associated mutations. Here, Lys86 and Arg351 tended to restore the interactions typical of state III (Figure 3B). Indeed, Lys86 interacted with T5 in state III and with T6 in state IV in the WT systems. Here, Lys86 increased interactions with T5 and decreased those with T6. Similarly, Arg351 lost its interactions with T7 in favor of T6. By contrast, Arg111 and Arg256 showed an opposite behavior. Arg111 decreased its WT interactions with T7 in favor of T9, and Arg256 left T9 for T10. Finally, Arg377 lost all the contacts with the primer strand in favor of T2. Interestingly, no residues that increased interaction with DNA relative to WT were found in state IV.

**Figure 4:**
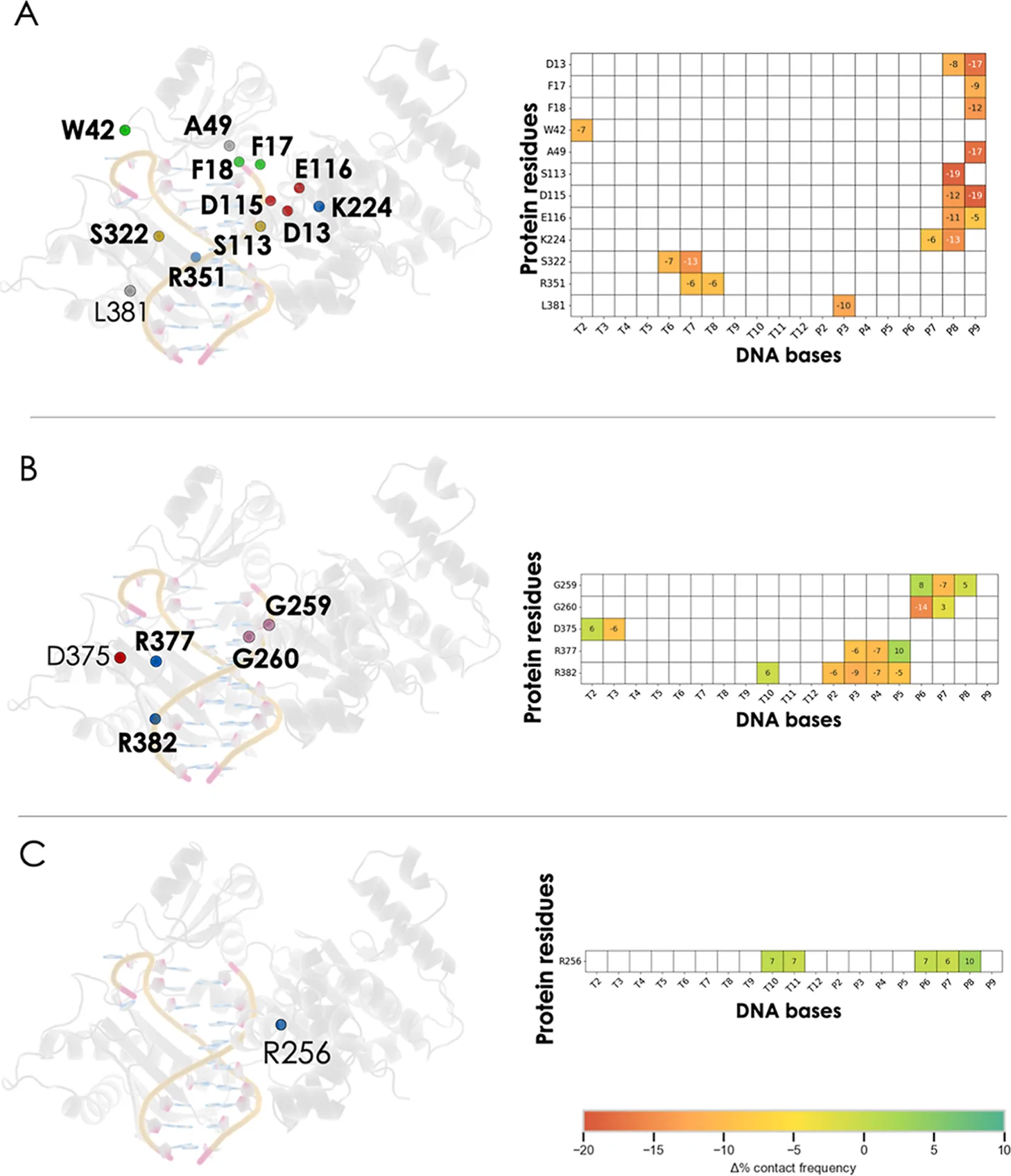
Structural and contact analysis of residues affected by Polη mutations in state III. Panels A, B, and C show residues whose protein–DNA contacts decrease (A), change (B), or increase (C) relative to wild-type (WT). In each panel, the top subpanel displays the protein–DNA complex in cartoon representation, with affected residues highlighted as spheres. Spheres are colored according to the chemical properties: positively charged (blue), negatively charged (red), aromatic (green), hydrophobic (white), aliphatic (pink), and special (yellow). The right subpanel shows the corresponding heatmap of Δ% of the contacts per residue–base pair, with DNA bases on the *x*-axis and protein residues on the *y*-axis. The shared colorbar indicates the magnitude and direction of change (red: negative Δ%, white: changes in Δ% within ±5%, green: positive Δ%).

Overall, these observations indicated that distinct XP-V mutations, although chemically and spatially diverse, converged on a common effect: perturbing protein–DNA contacts. This convergence provided a mechanistic rationale for the diverse phenotypes observed in XP-V variants and motivated our closer examination of the mutation-induced changes at the protein–DNA interface to identify common features shared across variants.

### XP-V Mutations Differentially Modulate DNA Binding Energetics and Allosteric Dynamics

After identifying a common perturbation pattern in protein–DNA contacts, we investigated the impact of both disease-associated mutations on DNA binding and the associated dynamical rearrangement confined within the enzyme. MM/PBSA calculations in state III revealed that energetic differences were already encoded prior to DNA translocation (Table S2).^38^ While the WT system exhibits a near-neutral binding free energy in state III (Δ*G* = −1 ± 1 kcal/mol), disease-associated mutations displayed heterogeneous effects, as reflected by the corresponding ΔΔ*G* values relative to WT (Table S2). These ranged from significant destabilization of the DNA-bound state (e.g., Arg111His, ΔΔ*G* = +32 ± 2 kcal/mol) to its premature stabilization (e.g., Thr122Pro, ΔΔ*G* = −53 ± 2 kcal/mol). This was also true for DNA binding in the state IV (WT , Δ*G* = −34 ± 1 kcal/mol), where disease-associated variants again spanned a wide energetic range, from destabilization (Val266Asp, ΔΔ*G* = +41 ± 2 kcal/mol) to stabilizing effects (Gly295Arg, ΔΔ*G* = −14 ± 2 kcal/mol). Overall, these mutations exhibited a consistent energetic trend, preferentially stabilizing the pre-translocation state while destabilizing the post-translocation state (Table S2). This pattern suggested a possible reshaping of the relative free-energy landscape along the translocation pathway, potentially increasing the energetic cost of progressing toward the post-translocation configuration.

Indeed, in the WT, translocation was associated with a substantial stabilization of DNA binding (ΔΔ*G*_post–pre_ = −33 ± 2 kcal/mol), suggesting a more stable post-translocation state (Table S3). However, several mutations significantly altered this energetic transition. For instance, Arg361Ser partially preserved the WT trend (ΔΔ*G*_post–pre_ = −32 ± 2 kcal/mol), indicating limited retention of the WT-like energetic profile. In contrast, Thr122Pro strongly disrupted the energetic profile for DNA translocation, leading to a marked inversion of translocation-associated stabilization (ΔΔ*G*_post–pre_ = +42 ± 2). On the other hand, Arg111His exhibited a pronounced stabilization of the WT energetic trend (ΔΔ*G*_post–pre_ = −55 ± 2 kcal/mol). Overall, these results demonstrated that XP-V–associated mutations did not simply modulate absolute binding affinity but can also differentially reshaped the energetic profile as it transits from the pre- to the post-translocation state, thereby likely perturbing the progression of the catalytic cycle (Tables S2–S3).

Distance-fluctuation (DF) analysis was next employed to characterize how mutations reshape the dynamical network within the enzyme (see Materials and Methods).^39^ By comparing mutant systems to the WT reference (ΔRMS = RMS_mut_ – RMS_WT_), we identified mutation-induced redistributions of flexibility across functionally relevant subdomains, including the finger, palm, thumb, and LF subdomains (Figure S32). In the WT system, state IV exhibited a relatively balanced, well-organized fluctuation pattern across the catalytic core, consistent with a coordinated interaction network that stabilizes DNA binding. Ala117Pro induced increased fluctuations in state III, particularly between the LF subdomain and the catalytic core, despite the mutation not being in this region, suggesting long-range allosteric propagation. In state IV, only mild increases in flexibility were observed between the palm and thumb subdomains. Thr122Pro showed a similar but stronger effect: extensive increases in fluctuations were already present in state III, while in state IV a pronounced destabilization was observed in the thumb subdomain, indicating disruption of the functional postbound network. Arg93Pro perturbed interdomain communication in state III, with increased fluctuations between thumb–LF and thumb–finger interfaces, while the state IV remained relatively close to WT. In contrast, Arg111His showed mild pre-translocation perturbations but strong post-translocation effects, with increased fluctuations between finger and LF and across the catalytic core, indicating destabilization of the communication network after translocation. Although located in the LF subdomain, Arg361Ser did not significantly affect local LF fluctuations. Instead, increased fluctuations were observed at the LF–core interface, suggesting that the mutation primarily affects interdomain coupling rather than local stability. Overall, the system remained relatively rigid, with limited perturbations elsewhere. Gly263Val induced moderate increases in fluctuations at the thumb–finger and thumb–LF interfaces in state III, which were partially retained but attenuated in state IV, suggesting partial stabilization upon translocation. Val266Asp exhibited strong increases in fluctuations in the pre-translocation state, both locally and across distant interfaces, including finger–thumb and finger–LF regions, indicating a global disruption of the dynamical network. In the post-translocation state, this effect was reduced locally but persisted at long-range interfaces, suggesting incomplete recovery of the interaction network. In contrast, Gly265Arg showed more localized increases in fluctuations near the mutation site in both states. However, in state IV, pronounced increases emerged between the LF subdomain and the catalytic core, indicating a redistribution of dynamical stress toward interdomain interfaces rather than a global destabilization.

It is reassuring that a qualitative correlation is observed between MM/PBSA energetic changes (Tables S2 and S3) and distance fluctuation (DF) profiles (Figure S32). Mutations that strongly affect DNA binding free energy, such as Gly295Arg, also induced widespread redistribution of interdomain fluctuations, consistent with a marked reorganization of the dynamical coupling between the thumb, finger, and LF subdomains. In these cases, the perturbation extended beyond local regions and propagated across the protein–DNA interface. In contrast, mutations associated with more moderate energetic effects, such as Gly263Val and Arg361Ser, primarily induced localized or interface-restricted changes in fluctuation patterns, suggesting partial preservation of the global dynamical organization with selective reweighting of specific communication pathways. Overall, these results support a model in which Polη function is modulated by a distributed dynamical network connecting DNA-binding regions and catalytic subdomains. Rather than inducing a complete collapse of this network, XP-V–associated mutations appear to differentially redistribute interdomain flexibility while concurrently altering the DNA binding free energy, providing a mechanistic link between energetic and dynamical perturbations across functional states.

## Discussion

Recently, time-resolved X-ray structures captured the main steps of the Polη catalytic cycle.^8,22^ While these structures clarify how the WT enzyme catalyzes nucleotide synthesis and translocates along DNA, the mechanistic effects of cancer-associated mutations in Polη remained unclear.^18^ Here, we employed equilibrium MD simulations of eight XP-V–associated protein mutations identified in patients to assess their impact on active-site geometry, ion coordination, and DNA binding throughout the translocation step.^26–29^ This subset of clinical mutations comprises only missense mutations associated with skin cancer (Table in Figure 2). We performed multiple replicas of pre-translocation (state III) and post-translocation (state IV) states for each of the eight single-point pathological mutations. Remarkably, the two mutations located in close vicinity to the active site (Ala117Pro and Thr122Pro) induced subtle but functionally significant distortions within the palm subdomain. By perturbing the β-strand adjacent to the catalytic triad (Asp15, Asp115, Glu116), these variants alter active-site geometry and partially impair sodium coordination at the A-site (Figure S9), particularly in the post-translocation state. Importantly, Thr122Pro and Ala117Pro retained much of the local active-site geometry, suggesting that the local structural framework could be compatible with metal-ion coordination, although the Mg^2+^ was not explicitly simulated (Figure S11). However, this apparent local structural preservation did not translate into full functional recovery of the translocation step. Instead, it was accompanied by persistent DNA misalignment and a reduced stabilization of the state IV, suggesting an impairment in the energetic and allosteric coupling required for DNA translocation. In particular, Ala117Pro induced a marked alteration of the energetic coupling between states III and IV, with a strong stabilization of state III and a concomitant destabilization of state IV. This results in a pronounced reduction of the free-energy gain associated with the transition, without a full inversion of the energetic landscape. Consistently, this perturbation was accompanied by a loss of conformational fluctuations in the post-translocation state, suggesting a rigidification of the structural ensemble. By contrast, Thr122Pro showed an inversion in the energetic landscape of the translocation process, leading to a complete reversal of the WT free-energy difference between the two states (Table S3). Clinically, these variants have been reported with relatively milder phenotypes, although no direct causal link can be inferred from the MD data.

On the other hand, mutations affecting the protein–DNA interface (Arg93Pro, Arg111His, and Arg361Ser) produced more extensive structural and functional consequences.^12,15,18,40^ Arg93Pro disrupted the steric gate and compromised template-strand stability.^41–46^ By contrast, the active site of the Arg93Pro variant stably coordinated sodium ions (Figure S14). Arg111His altered DNA-template contacts and increases local conformational flexibility. The interaction with the linker residue Arg313 (4.7 ± 0.8 Å) prevented proper DNA coordination by the enzyme. Although both variants recruited sodium ions to compensate for the loss of positive charge (Figure S15), these interactions failed to fully restore proper DNA alignment (Figures S3, S4 and S23, S24). Although Arg361Ser did not directly contact DNA, its effect destabilized the LF helix and the molecular splint that guides the template strand, causing the persistent misalignment of the thumb subdomain and consequent loss of DNA binding (Figure S17). Consistent with these structural observations, MM/PBSA calculations (Table S2) and DF network analysis (Figure S32) reveal distinct but convergent energetic and dynamical signatures across interface mutants. Arg93Pro mildly reduces the stabilization of the state IV compared to the WT (ΔΔ*G* = +12 ± 2 kcal/mol), while maintaining state III binding affinity. Despite minimal structural reorganization (Figure S32), the mutation leads to a measurable reduction in the free-energy difference between the two states, indicating a mild attenuation of the WT driving force for translocation. By contrast, Arg111His significantly destabilizes state III while only moderately affecting state IV (Table S2). Notably, this energetic redistribution occurs without detectable changes in the local dynamical fluctuations, indicating that the structural integrity of both states remains largely preserved (Figure S32). As a consequence, the free-energy difference between the two states is markedly increased relative to the WT, indicating reduced stabilization in state III. This is accompanied by enhanced local flexibility and disruption of Polη–template contacts, suggesting a failure to maintain proper coupling between the linker region and the DNA scaffold. On the other hand, Arg361Ser has negligible effects on the free-energy landscape and the system’s conformational dynamics. The free-energy difference between states III and IV remains essentially unchanged relative to the WT, indicating that the overall thermodynamic driving force for translocation is preserved. Consistently, no significant alterations are observed in the local or global fluctuation patterns in either state, suggesting that Arg361 does not contribute to the electrostatic interaction network governing DNA binding and translocation coupling.

A similar propagation of structural perturbations was observed for thumb subdomain mutations (Gly263Val, Val266Asp, and Gly295Arg). Gly263Val introduced steric hindrance within a conserved hydrophobic pocket, whereas Val266Asp and Gly295Arg weakened the electrostatic interactions with the DNA duplex.^21^ Notably, these effects persisted across both states, indicating a mutation-independent mechanism by which thumb destabilization can compromise DNA binding and primer translocation (Figures 3, 4 and 5). Gly263Val induces only minor perturbations in state III, resulting in a slight stabilization without detectable changes in the overall dynamical fluctuations of either state. The state IV remains essentially unaffected, and the free-energy difference between the two states is only marginally reduced relative to the WT. By contrast, Val266Asp leads to a pronounced destabilization of state IV, while leaving state III largely energetically unperturbed. This asymmetry is accompanied by increased local fluctuations in state III, whereas state IV displays reduced structural stability consistent with a loss of conformational integrity. Therefore, the free-energy difference between the two states is markedly altered relative to the WT, indicating a substantial impairment of the transition toward the post-translocation ensemble. These results suggest that Val266 is involved in maintaining the structural stability of the post-translocation configuration. Finally, Gly295Arg induces moderate stabilization of both states, resulting in a reduced free-energy difference between them relative to the WT. Despite this overall energetic stabilization, the state IV exhibits markedly enhanced conformational fluctuations, indicating a loss of dynamical coherence. In contrast, state III is comparatively less affected by structural fluctuations. Together, these results indicate that thumb-subdomain mutations primarily affect the mechanical transmission of conformational changes during DNA translocation, perturbing structural contacts and dynamical coupling in a mutation-dependent manner. Collectively, these effects weaken the efficiency of post-translocation state formation.

**Figure 5:**
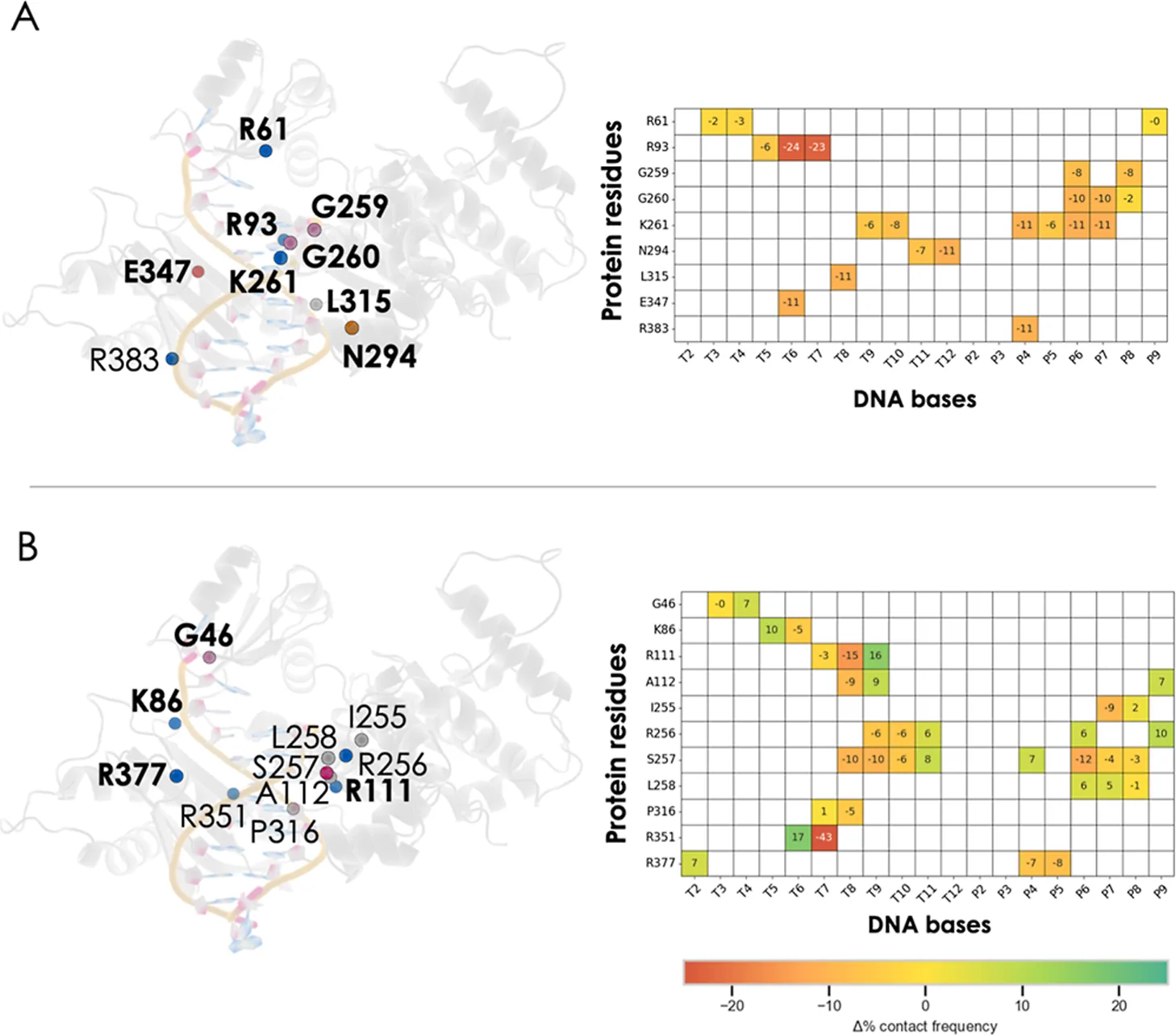
Structural and contact analysis of residues affected by Polη mutations in state IV. Panels A and B show residues whose protein–DNA contacts decrease (A), or change (B) relative to WT. No residues exhibited an increase in contacts in state IV, so panel C is not shown. In each panel, the top subpanel displays the protein–DNA complex in cartoon representation, with affected residues highlighted as spheres. Spheres are colored according to the chemical properties: positively charged (blue), negatively charged (red), aromatic (green), hydrophobic (white), aliphatic (pink), and special (yellow). The right subpanel shows the corresponding heatmap of Δcontacts per residue–base pair, with DNA bases on the *x*-axis and protein residues on the *y*-axis. The shared colorbar indicates the magnitude and direction of change (red: negative Δ%, white: changes in Δ% within ±5%, green: positive Δ%).

To further investigate how pathogenic mutations can modify the protein–DNA interface, we identified all protein residues in close contact (4.5 Å) with the DNA in the WT system and compared them across all mutants (see Materials and Methods). We immediately observed that mutations in different regions of the protein resulted in a generalized rearrangement of the enzyme. Notably, even mutations not in close contact with the DNA duplex perturbed DNA anchoring. Thus, we focused our analysis on finding a rearrangement in protein–DNA interactions. Despite the mutation-specific nature of these local effects, analysis of protein–DNA contacts revealed a striking convergence across XP-V variants. In state III, 31% of native contacts were perturbed, with more than half shared by at least seven of the eight mutants, whereas in state IV, this fraction increases to 43%, with 21% of affected residues being positively charged (Figures 4 and 5). Residues exhibited distinct behaviors (loss of contacts, redistribution toward neighboring bases, or compensatory interactions), but these perturbations consistently persisted across both states. This persistence in structural and dynamical perturbations in both states III and IV suggests that XP-V–associated mutations can impact DNA translocation, while effects on other catalytic intermediates remain to be investigated. Our simulations also showed that XP-V variants differ in the extent to which they perturb the protein–DNA interface. Variants affecting the molecular splint, DNA translocation, or distal structural elements (Arg93Pro, Arg111His, Arg361Ser, Gly263Val, Val266Asp, Gly295Arg) consistently induce broader rearrangements of the protein–DNA interface, accompanied by a reduction or reshaping of the free-energy stabilization associated with the state IV and a marked redistribution of interdomain fluctuations. In contrast, mutations primarily impacting the catalytic site (Ala117Pro, Thr122Pro) display more localized structural effects. Yet, they still exhibit an attenuation of the energetic gain upon translocation and evidence of altered allosteric coupling, as revealed by MM/PBSA (Table S2) and DF analyses (Figure S32). These observations provide a molecular fingerprint of XP-V variants, linking the spatial origin of the mutation to distinct regimes of structural perturbation, energetic remodeling, and dynamical network disruption affecting protein–DNA interactions and active-site communication.

Collectively, our results support a multilayered model of DNA translocation in Polη, in which distinct structural regions contribute in a functionally specialized but interconnected manner. The active site modulates the energetic coupling between states III and IV, acting as a primary determinant of the directionality and magnitude of the free-energy landscape, as exemplified by the distinct behaviors of Ala117Pro and Thr122Pro. In parallel, residues at the protein–DNA interface regulate the electrostatic environment and tune the probability and efficiency of state transitions without directly altering the conformational framework, as observed for Arg93Pro, Arg111His, and Arg361Ser. Finally, the thumb subdomain governs the mechanical transmission of conformational changes and the structural integrity of state IV, with mutations inducing either local gating defects or energy-dynamics decoupling effects, as in Val266Asp and Gly295Arg. Despite their distinct structural origins, all mutations converge on a common outcome: disruption of the energetic and dynamical coupling required for efficient DNA translocation. These findings establish that Polη translocation is not governed by a single molecular switch, but emerges from the coordinated interplay of energetic, electrostatic, and mechanical regulatory layers distributed across the enzyme–DNA complex.

Finally, a structural comparison with another Y-family member, Polκ, revealed that several Polη residues affected by XP-V mutations occupy analogous positions in Polκ where cancer-associated mutations have been reported (Arg361Ser/Arg512Trp, Thr122Pro/Thr102Ala, Arg93Pro/Arg175Gln).^47,48^ This conservation underscores the functional importance of these sites across Y-family polymerases and highlights the evolutionary significance of the collective and functional dynamics of the thumb, LF, and DNA-binding regions in maintaining efficient polymerase catalysis.

## Conclusions

In summary, we analyzed eight XP-V–associated missense mutations and showed that they induce distinct but convergent perturbations of the DNA-bound Polη system across pre- and post-translocation states. These mutations affect structurally diverse regions of the enzyme yet consistently disrupt DNA binding and translocation through a combination of local structural rearrangements, long-range allosteric effects, and altered interdomain communication. By integrating molecular dynamics simulations with free energy semiquantitative calculations and distance-fluctuation network analysis, we show that pathogenic variants not only perturb local active-site or interface geometry but also impact the binding free energy. Several mutations reduce or alter the free-energy stabilization associated with the post-translocation state (state IV), while simultaneously inducing a redistribution of dynamical couplings across the thumb, little finger, and finger subdomains. Importantly, these effects are mutation-dependent in their structural origin but converge functionally toward a common outcome: impaired coordination between DNA binding, translocation, and proper catalytic-site organization. This establishes a unified mechanistic framework linking XP-V missense mutations to defective Polη function and provides a molecular explanation for the heterogeneous clinical phenotypes observed in XP-V patients. These findings provide a foundation for future investigations of Polη function in the presence of DNA lesions.

## Materials and Methods

### Structural Models

Eight mutant models, namely the Arg93Pro, Arg111His, Ala117Pro, Thr122Pro, Gly263Val, Val266Asp, Gly295Arg, and Arg361Ser mutations of human DNA polymerase η (Polη) were prepared, starting from the two WT models described in Visigalli et al.^18^ Here, Polη is in complex with the TpCpGpCpApGpTpApTpTpApC template, the ApGpCpGpTpCpApT primer, and lacks the catalytic Mg^2+^ ions. Each single-point mutation was introduced by substituting the corresponding residues in the wild-type (WT) structures using MODELLER 10.3.^49,50^ The models’ stereochemical quality was assessed using PROCHECK.^51^ Complexes were solvated with water in a rhombic dodecahedral simulation cell using tleap from AmberTools25.^52^ Monovalent ions Na^+^ and Cl^–^ were added to the solvent to neutralize the system and achieve the physiological ionic concentration of 150 mM.^18,52^ Each system consisted of ∼ 70,000 atoms.

### Molecular Dynamics

The MD simulations were performed using the GPU version of the pmemd module of AMBER 24, running on the JUWELS BOOSTER supercomputer.^53–56^ The AMBER-ff14SB,^57^ bsc1,^58,59^ Li-Merz,^60^ and TIP3P^61–63^ force fields were used for the protein, DNA, ions, and water, respectively. Long-range electrostatic interactions were treated using the Particle Mesh Ewald (PME) method with a cutoff for the real part of 10 Å.^55,64,65^ The Van der Waals interactions were truncated at 10 Å. The SHAKE algorithm^62^ was applied to constrain all bonds involving hydrogen atoms. A 2 fs time step was used.^66^ Periodic boundary conditions in the three dimensions of the Cartesian space were applied to the systems. NPT conditions were achieved by using a Langevin thermostat^67^ (except for step VII below, for which we used the Andersen-like thermostat^68^) and a Monte Carlo barostat with a target pressure of 1 atm.^69^

Our simulation protocol consists of the following steps: (I) energy minimization of the groups reconstructed using MODELLER.^49,50^ Specifically, we applied a harmonic restraint of 100 kcal mol^–1^ Å^–2^ for 10,000 steps, using the steepest descent algorithm, followed by a conjugate gradient algorithm for 10,000 steps. (II) NPT solvent molecular dynamics for 20 ns with a 2 fs time step, at a temperature of 310 K and a pressure of 1 atm.^67,69^ (III) Side-chain energy minimization. We applied a harmonic restraint of 100 kcal mol^–1^ Å^–2^ to the protein and DNA backbone using the procedure described in (II). (IV) Energy minimization of the whole system, using the same procedure as (II). (V) 0.1 ns NVT MD at 100 K for 100 ps, fixing the protein and DNA backbone with a harmonic restraint of 100 kcal mol^–1^ Å^–2^. (VI) Then, we gradually increased the temperature from 100 to 310 K by 2 ns NPT MD at 1 atm.^69^ (VII) 3 ns NPT MD at 310 K and 1 atm, allowing it to equilibrate at the target temperature without any restraints. (VIII) For each variant and state, three independent replicas of approximately 1 μs each were generated (NPT; 310 K; 1 atm). Simulations were carried out for both pre-translocation (III) and post-translocation (IV) states of the catalytic cycle. For reference, we compared the variants to our simulations of the WT enzyme,^18^ which establishes baseline features of the catalytic triad, DNA-binding residues, and subdomain architecture.^45,46^ All simulations reached stable conformational ensembles, as assessed by the root-mean-square deviation (RMSD) of protein and DNA (Figures S1–S8).

### Common Features Measurement

To identify convergent effects of XP-V–associated mutations on Polη–DNA interactions, we analyzed protein–DNA contacts across WT and mutant trajectories (∼3 μs per system, 3 independent replicas). Contacts were defined as any protein residue heavy atom within 4.5 Å of a DNA base heavy atom. For each residue–base pair, the contact frequency was calculated as the fraction of frames in which the contact is present over the trajectory using the native contacts function implemented in CPPTRAJ.^52^ Residues showing changes in contacts relative to the WT were further classified according to the nature of the perturbation. Some residues exhibited a decrease in contact frequency (<5%), reflecting a loss of interactions. Others maintained a comparable overall contact frequency (±5%) but redistributed their contacts among different DNA bases, indicating a shift in the interaction pattern. Finally, a few residues showed an increase in contact frequency (>5%), representing the formation of new interactions. Common features across the mutant ensemble were defined by identifying residues affected in at least 7 of the 8 variant systems. For each residue, the fraction of mutants exhibiting a given type of change was calculated, and residues were annotated by chemical property (positively charged, negatively charged, polar, or hydrophobic) to evaluate the roles of electrostatic and structural factors in the observed perturbations. This analysis was performed separately for state III and state IV ensembles, enabling the identification of both state-specific and state-independent mechanistic trends induced by XP-V–associated mutations.

### MM/PBSA Free Energy Calculations

Binding free energies were estimated using the MM/PBSA approach as implemented in the Python script MMPBSA.py of the AmberTools.^38^ Trajectories from equilibrium MD simulations of states III and IV were first processed using CPPTRAJ to remove solvent and ions, retaining only protein and DNA atoms.^52^ For each system, evenly spaced snapshots were extracted from the production trajectories after equilibration. Binding free energies were computed for complex, receptor, and ligand states as

$$\Delta G_{binding}=G_{binding}-\left(G_{protein}+G_{DNA}\right)$$

Poisson–Boltzmann (PB) implicit solvent models were employed to estimate solvation contributions. Entropic contributions were not explicitly included, and thus, the results represent relative binding free energy differences between WT and mutant systems. Final values were averaged over all frames.

### Distance Fluctuation Network Analysis

Distance fluctuation analysis was performed to characterize changes in residue–residue dynamical coupling upon mutation using the framework of coordination propensity as introduced by Morra et al.^39^ In this approach, mechanically coordinated motions are identified through low fluctuations in pairwise inter-residue distances over time. For each system, Cα-Cα distance matrices were computed from equilibrium molecular dynamics trajectories, using the default parameters. Distance fluctuation values were then evaluated and compared between WT and mutant systems to generate ΔDF matrices (ΔRMS-like representation), where positive values indicate increased dynamical decoupling and negative values indicate increased coordination relative to the WT system.

## Supplementary Material





## Data Availability

The structures used in this study were obtained from the RCSB Protein Data Bank (https://www.rcsb.org/). MODELLER v10.3 was used to model the two states, adding the missing residues to the protein (https://salilab.org/modeller/) and introducing each mutation to the corresponding model.^49,50^ PROCHECK was used to assess the stereochemical quality of the resulting models (https://www.ebi.ac.uk/thornton-srv/software/PROCHECK/).^51^ The tleap suite from AmberTools25 was used to create the system’s topology and to add the missing DNA bases.^52^ The GPU version of pmemd from AMBER24 was used to perform classical MD simulations (https://ambermd.org/).^50,53–55,70^ Binding free energy calculations were performed using the MM/PBSA approach implemented in the AMBER tools suite.^38,52^ GROMACS 2024.3 (DOI: 10.5281/zenodo.13456374) was used to calculate the radial distribution function in all systems.^71^ Bridge2 was employed to identify and measure the occupancy of the H-bond networks (https://github.com/maltesie/bridge2).^36,37^ In addition, distance fluctuation analysis was performed using the DF Analysis tool developed by the Colombo laboratory (https://github.com/colombolab/Distance-Fluctuation-DF-Analysis).^39^ All the structures in the figures have been produced using the educational version of PyMOL 3.1 (https://www.pymol.org).^72,73^ . Trajectories are available upon request, while AMBER input files (topology and coordinates) are available in AV’s GitHub repository (https://github/alevisigalli/PolETA_XPV).

## Notes

Clinical data details; further analysis of classical MD simulations and supplementary figures (PDF)
Input and output files used in classical MD simulations of XP-V pathological variant of Polη (ZIP)
