# Supplementary material for: Decoding Cancer-Associated Mutations in DNA Polymerase η through Atomistic Simulations
Source: J Chem Theory Comput. 2026 May 21;22(16):8566–78. doi: 10.1021/acs.jctc.6c00345 (PMC13523637; doi:10.1021/acs.jctc.6c00345)
Supplement: Supplementary file 1 [file ct6c00345_si_001.pdf]

Decoding Cancer-Associated Mutations in DNA Polymerase η through atomistic simulations

Alessia Visigalli[1,2], Paolo Carloni[*,2,3,4], and Marco De Vivo[*,1]

[1] Laboratory of Molecular Modeling & Drug Discovery, Istituto Italiano di Tecnologia, Via Enrico Melen 83, 16142, Genoa, Italy

2 Computational Biomedicine, Institute of Neuroscience and Medicine INM-9, Forschungszentrum Jülich GmbH, 52428 Jülich, Germany

3 Department of Physics, RWTH Aachen University, 52056 Aachen, Germany

4 JARA Institute: Molecular Neuroscience and Imaging, Institute of Neuroscience and Medicine INM-11, Forschungszentrum Jülich GmbH, 09042 Jülich, Germany

Corresponding authors:

Paolo Carloni: p.carloni@fz-juelich.de

Marco De Vivo, marco.devivo@iit.it

Supporting Information

TABLE OF CONTENTS

Supplementary Tables S1
Table S1. .... S2
Table S2. .... S4
Table S3. .... S5
Supplementary Figures S6
Figure S1. .... S6
Figure S2. .... S7
Figure S3. .... S8
Figure S4. .... S9
Figure S5. .... S10
Figure S6. .... S11
Figure S7. .... S12
Figure S8. .... S13
Figure S9. .... S14
Figure S10. .... S15
Figure S11. .... S16
Figure S12. .... S17
Figure S13. .... S18
Figure S14. .... S19
Figure S15. .... S20
Figure S16. .... S21
Figure S17. .... S22
Figure S18. .... S23
Figure S19. .... S24
Figure S20. .... S25
Figure S21. .... S26
Figure S22. .... S27
Figure S23. .... S28
Figure S24. .... S29
Figure S25. .... S30
Figure S26. .... S31
Figure S27. .... S32
Figure S28. .... S33
Figure S29. .... S34
Figure S30. .... S35
Figure S31. .... S36

Figure S32. .... S37
REFERENCES S38

Supplementary Tables

Category	Cell line	Genomic mutation	Aminoacids mutation	Protein subdomain	Symptoms
**Missense**	XP853VI	c.G278C	p.Arg93Pro	Finger	Aggressive
	XP36BR	c.G332A	p.Arg111His	Palm	Aggressive
	XPV3KO	c.G349C	p.Ala117Pro	Palm	ND
	XP5BI	c.A364C	p.Thr122Pro	Palm	No tumors
	XP11BR	c.G788T	p.Gly263Val	Thumb	Aggressive
	XP843VI	c.T797A	p.Val266Asp	Thumb	Mild
	XP603VI	c.G883A	p.Gly295Arg	Thumb	Aggressive
	XP57RO	c.G1083T	p.Arg361Ser	LF	Mild
	XPV8HM	c.A1766C	p.Lys589Thr	C-term	ND
	XP872VI	c.A2074G	p.Thr692Ala	C-term	Mild
**Truncations**	XPV6HM<br>XPV8HM	c.C725G	p.Ser242Stop	Thumb	ND<br>ND
	XP56RO	c.G890A	p.Trp297Stop	Thumb	None
	XP422VI	c.C907T	p.Arg303Stop	LF	Aggressive
	XPV11HM<br>XPV6KO	c.G916T	p.Glu306Stop	LF	ND<br>ND
	XP52RO<br>XP53RO<br>XPV2HM	c.C1066T	p.Arg356Stop	LF	Medium<br>Mild<br>ND
	XP86VI	c.C1561T	p.Gln521Stop	C-term	Mild
	XP1AB	c.C1543A	p.Thr-548Stop	C-term	Aggressive
**Deletions**	XP966VI<br>XP967VI	c.108-110del3	p.Val37del	Finger	Medium<br>Mild
	XP2DU<br>XP3DU<br>XP6DU	c.207delG	Fs69		ND<br>ND<br>Aggressive
	XP58RO<br>XP2DU<br>XP3DU<br>XP6DU<br>XP7BR<br>XP1AB	Del 224–226	p.Leu-75del	Finger	Aggressive<br>ND<br>ND<br>Aggressive<br>ND<br>ND
	XPV1HM<br>XPV7HM	c.G490T	p.(?)fs*192 or<br>p.(Gly134_Lys163del)	Palm	ND<br>ND

	XPV10HM<br>XPV1KO<br>XPV2KO<br>XPV5KO				ND<br>ND<br>ND<br>ND
	XP51VI	c.491-? 660+?del	p.Glu164Glyfs∗37	Palm	Medium
	XP819VI	c.661–483<br>764+159 del	p.Val221Profs∗2	Palm	Aggressive
	XP878VI	c.661-? 884+?del	p.Lys224Ilefs∗8	Palm	Medium
	XP888VI<br>XP968VI	c.672delA	p.Lys224Trpfs229	Palm	Mild<br>Mild
	XP28VI<br>XP62VI<br>XP546VI<br>XP865VI	c.1075-?<br>1244+?del	p.Asn359Valfs∗32	LF	Aggressive<br>Mild<br>Mild<br>Mild
	XP7DU<br>XP422VI	c.1222-<br>1225delACTT	p.Thr408Leufs∗36		ND<br>ND
	XPV12HM	c.1661delA	p.Gln554Leufs*31	C-term	ND
	XP603VI	c.1727 1728 delCT	p.Pro576Argfs∗3	C-term	Aggressive
	XP51VI<br>XP53RO<br>XP56RO	Del exon 5	Fs 163		Medium<br>Mild<br>None
	XP62VI<br>XP75VI<br>XP28VI	Del exon 10	Fs 358		Medium<br>Medium<br>Medium
Insertions	XP961VI	c.437insA	p.Tyr146Stop	Palm	Mild
	XP7BR	c.764ins?	Fs255		ND
	XP53RO	c.882_883insT	Fs 294		Mild
	XP28VI<br>XP127VI	c.1091_1092insC	Fs 364		Medium<br>Aggressive
	XP127VI	c.1093insC	p.Gln365Profs∗27	LF	Aggressive
	XP37BR	c.1668insC	Fs556		Mild
Splicing	XP606VI<br>XP737VI<br>XP968VI	c.-5+1 G >C	p.?		Medium<br>Mild<br>ND
Stop-loss	XP872VI	c.A2141G	p.714Trpext8	C-term	Mild

Table S1.

This table summarizes all reported mutations of DNA polymerase η (Polη) associated with Xeroderma Pigmentosum Variant (XP-V). For each mutation, the table reports the mutation category (e.g., missense, nonsense, or frameshift), the cell line or biological source in which it was identified, the genomic mutation, the corresponding amino acid substitution, the affected protein subdomain, and

the associated clinical symptoms. This overview enables a direct comparison between genetic alterations in Polη, their structural localization, and the resulting disease phenotypes.[1–4]

Mutations	$\Delta\Delta G_{pre} = (\Delta G_{mut} - \Delta G_{wt})_{pre}$ (kcal/mol)	$\Delta\Delta G_{post} = (\Delta G_{mut} - \Delta G_{wt})_{post}$ (kcal/mol)
Ala117Pro	-18 ± 2	+12 ± 2
Thr122Pro	-53 ± 2	+22 ± 2
Arg93Pro	0 ± 2	+12 ± 2
Arg111His	+32 ± 2	+10 ± 2
Arg361Ser	-6 ± 2	-5 ± 2
Gly263Val	-4 ± 2	0 ± 2
Val266Asp	0 ± 2	+41 ± 2
Gly295Arg	-24 ± 2	-14 ± 2

Table S2.

MMPBSA-derived relative binding free energy differences (ΔΔG) for selected Polη mutations in pre-translocation (III) and post-translocation (IV) conformations.[5] Energies are reported as $\Delta\Delta G = \Delta G_{mut} - \Delta G_{wt}$ (kcal/mol), averaged over the sampled trajectories with associated SEM uncertainties. Positive (negative) values indicate reduced (increased) binding affinity of the mutant compared to the wild type.

Mutations	$\Delta\Delta G = (\Delta G_{post} - \Delta G_{pre})$ (kcal/mol)
Wild-Type	-33 ± 2
Ala117Pro	-3 ± 2
Thr122Pro	+42 ± 2
Arg93Pro	-21 ± 2
Arg111His	-55 ± 2
Arg361Ser	-32 ± 2
Gly263Val	-29 ± 2
Val266Asp	+8 ± 2
Gly295Arg	-23 ± 2

Table S3.

Differences in mutation-induced binding free energy changes between post-translocation (IV) and pre-translocation (III) states of Polη, defined as $\Delta\Delta G = \Delta G_{post} - \Delta G_{pre}$ (kcal/mol).[5] Positive values indicate a relative increase in mutation-induced destabilization upon translocation, whereas negative values indicate a reduction of the mutational effect in the post-translocation state or a relative stabilization compared to the pre-translocation state. Values are reported as mean ± SEM.

Supplementary Figures

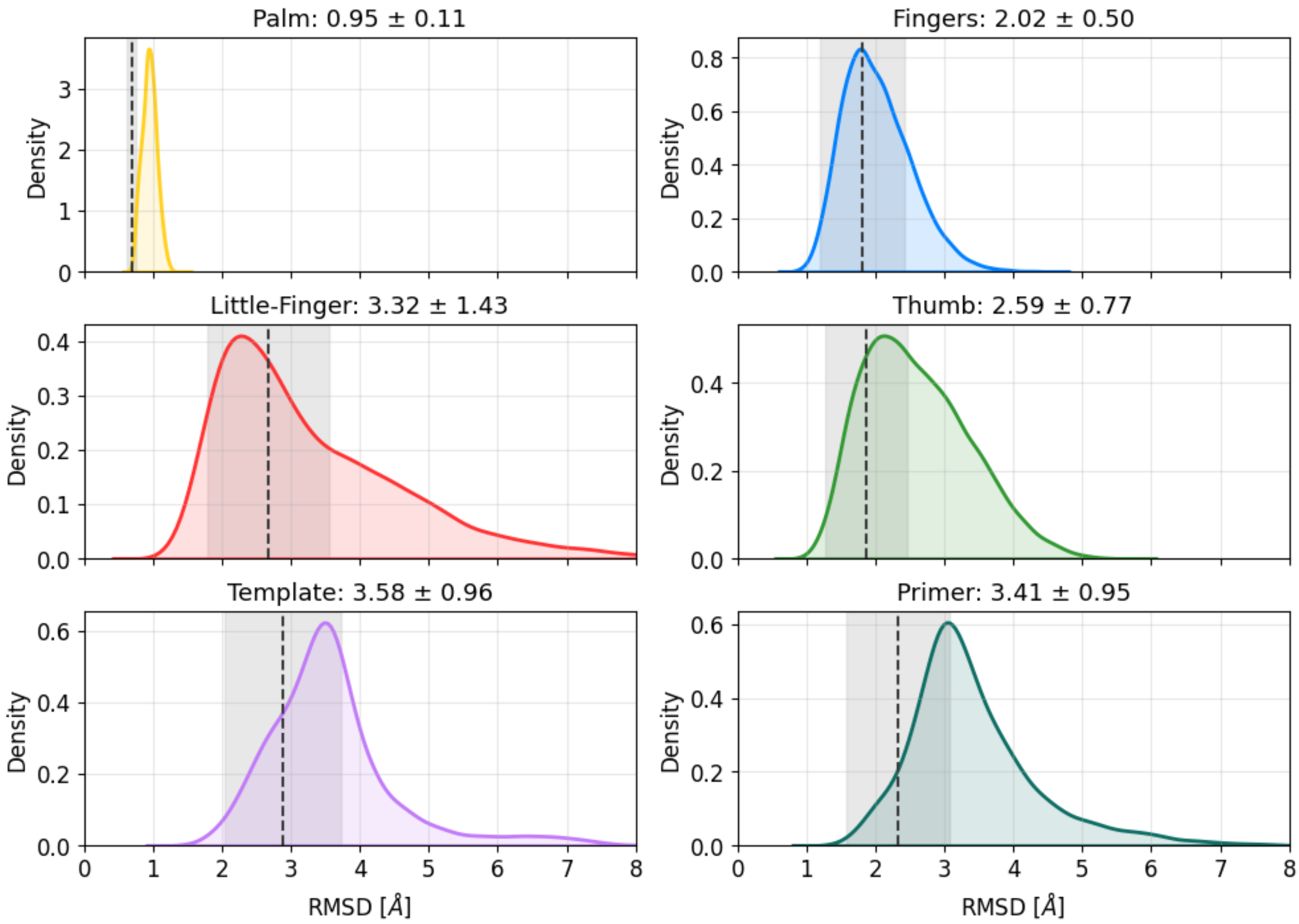

Figure S1.

Root-Mean-Square Deviation (RMSD) of the Ala117Pro variant in state III. (top) Each plot referred to the Palm (gold), Finger (blue), Thumb (green), LF (red), Template (pink), and Primer (teal). The black dashed line and the gray stripe represent the mean value and the standard deviation in the wild-type system.[6]

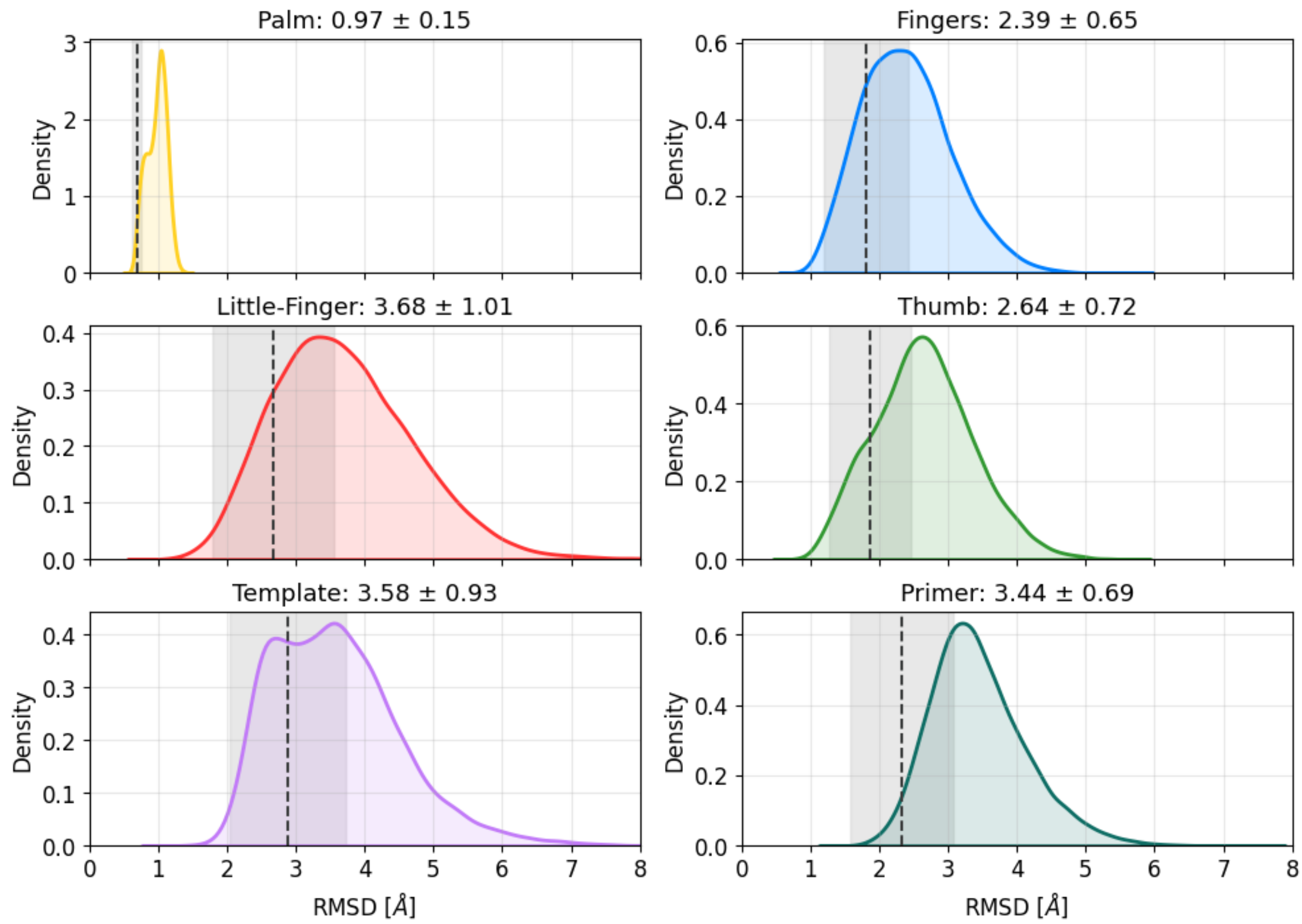

Figure S2.

Root-Mean-Square Deviation (RMSD) of the Thr122Pro variant in state III. (top) Each plot referred to the Palm (gold), Finger (blue), Thumb (green), LF (red), Template (pink), and Primer (teal). The black dashed line and the gray stripe represent the same quantity as those of Figure S1. [6]

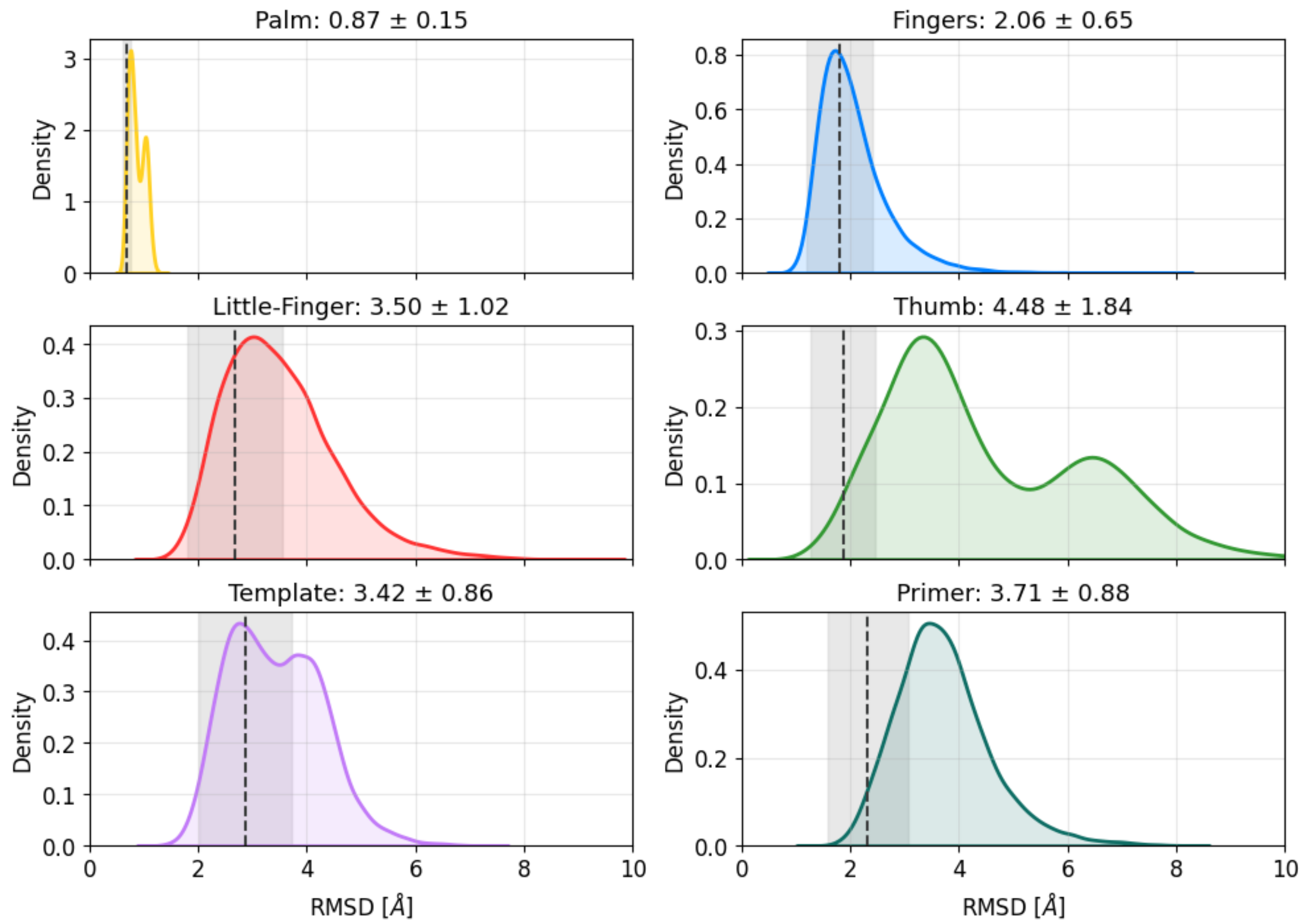

Figure S3.

Root-Mean-Square Deviation (RMSD) of the Arg93Pro variant in state III. (top) Each plot referred to the Palm (gold), Finger (blue), Thumb (green), LF (red), Template (pink), and Primer (teal). The black dashed line and the gray stripe represent the same quantity as those of Figure S1. [6]

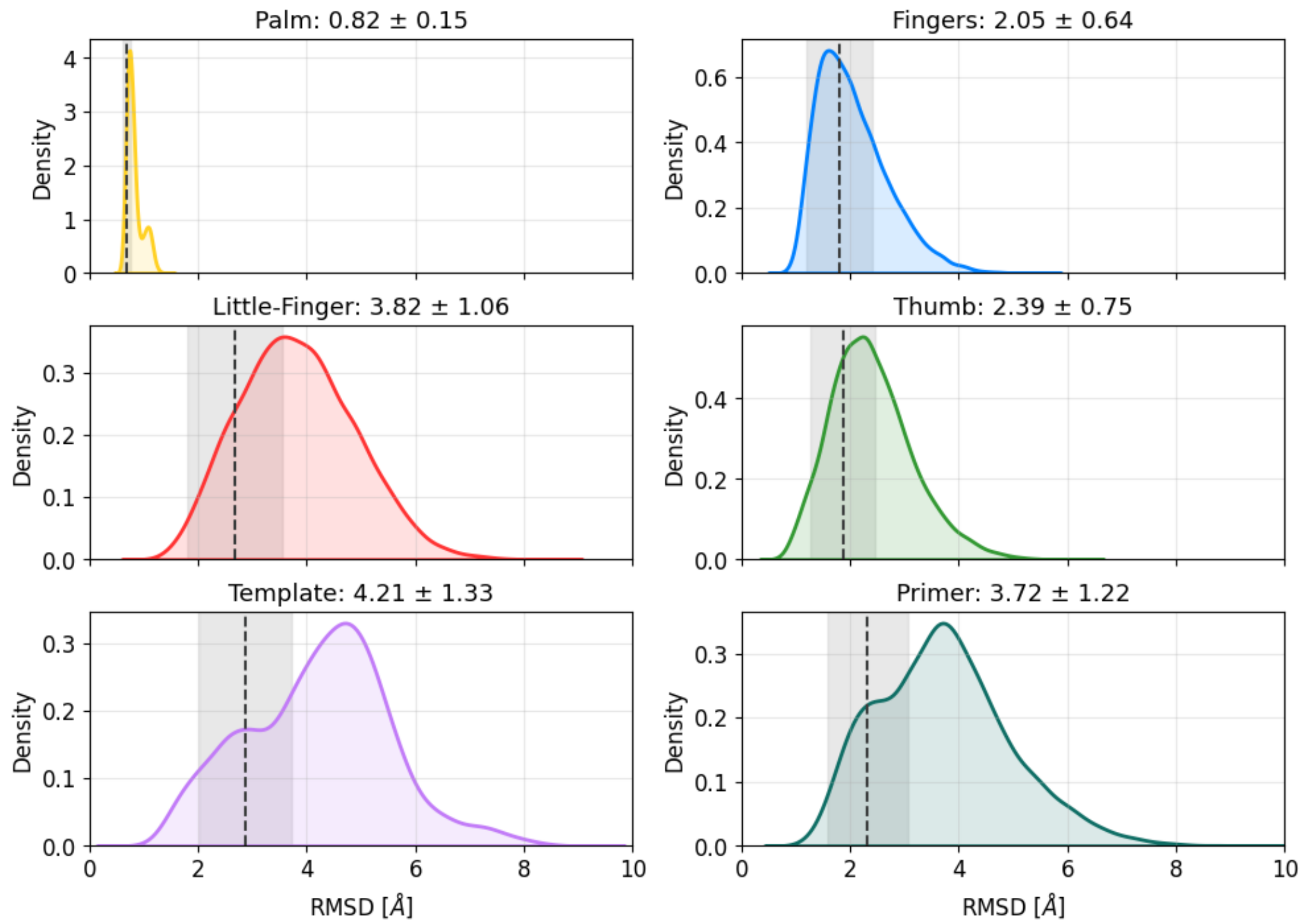

Figure S4.

Root-Mean-Square Deviation (RMSD) of the Arg111His variant in state III. (top) Each plot referred to the Palm (gold), Finger (blue), Thumb (green), LF (red), Template (pink), and Primer (teal). The black dashed line and the gray stripe represent the same quantity as those of Figure S1.[6]

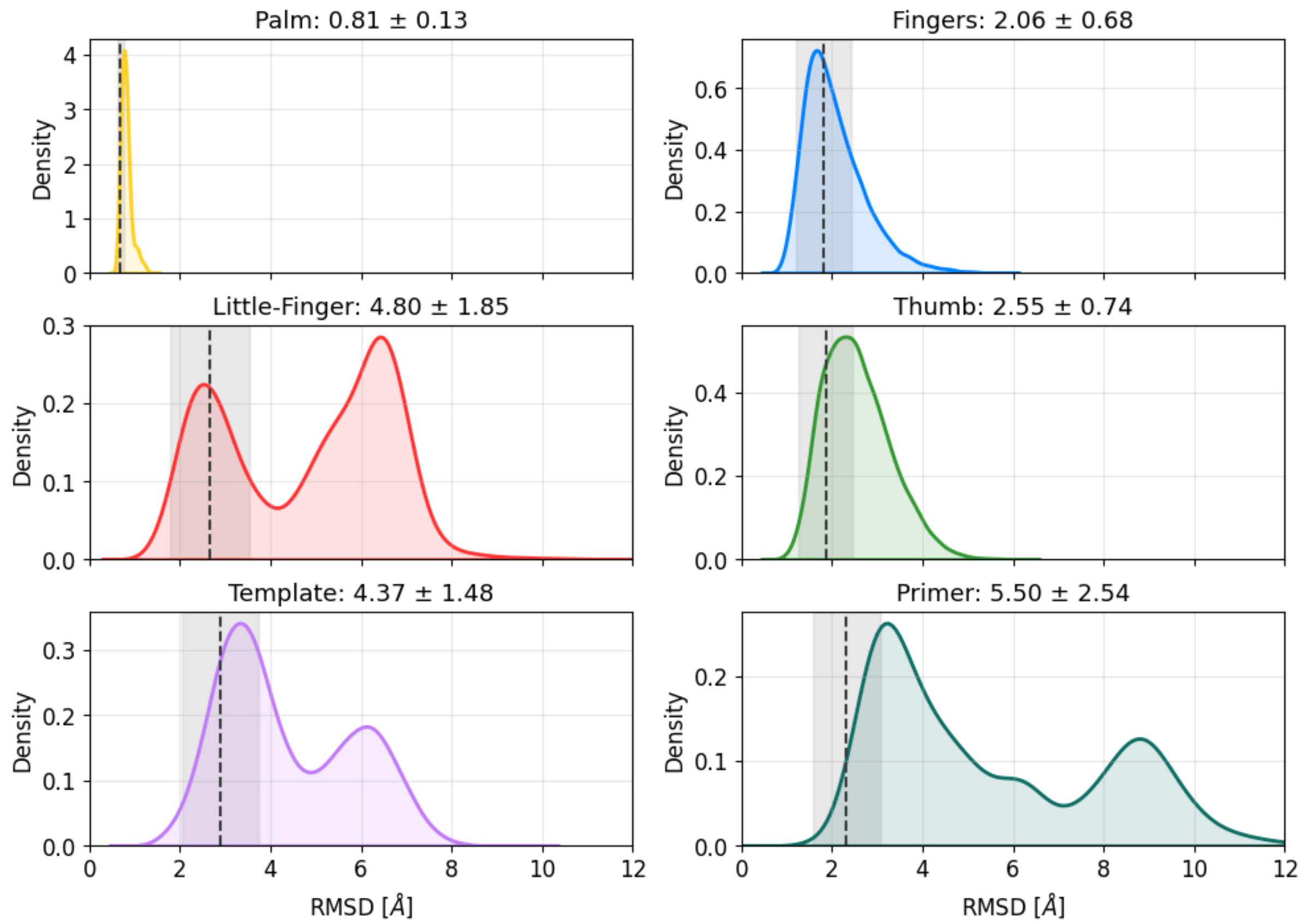

Figure S5.

Root-Mean-Square Deviation (RMSD) of the Arg361Ser variant in state III. (top) Each plot referred to the Palm (gold), Finger (blue), Thumb (green), LF (red), Template (pink), and Primer (teal). The black dashed line and the gray stripe represent the same quantity as those of Figure S1.[6]

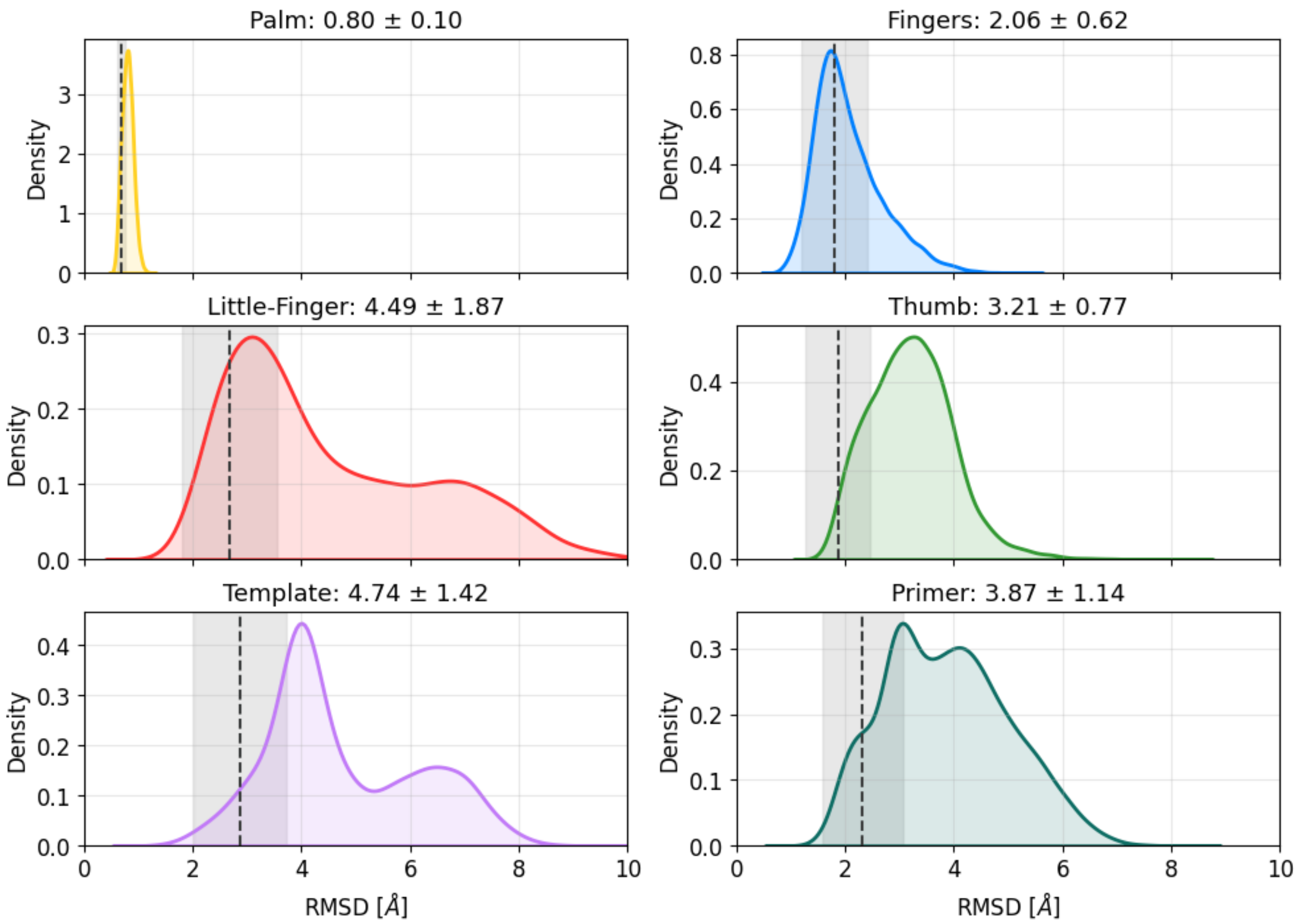

Figure S6.

Root-Mean-Square Deviation (RMSD) of the Gly263Val variant in state III. (top) Each plot referred to the Palm (gold), Finger (blue), Thumb (green), LF (red), Template (pink), and Primer (teal). The black dashed line and the gray stripe represent the same quantity as those of Figure S1.[6]

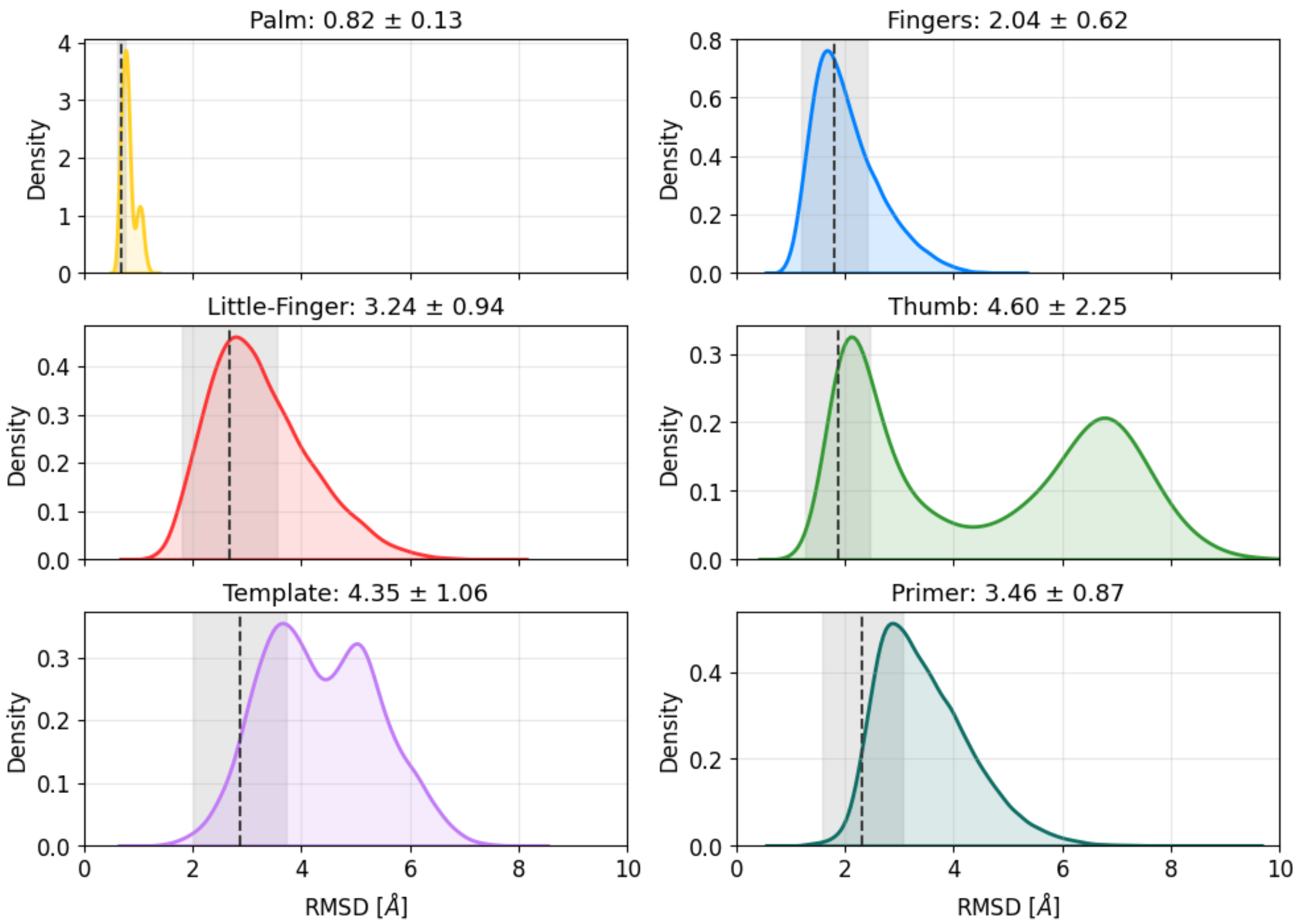

Figure S7.

Root-Mean-Square Deviation (RMSD) of the Val266Asp variant in state III. (top) Each plot referred to the Palm (gold), Finger (blue), Thumb (green), LF (red), Template (pink), and Primer (teal). The black dashed line and the gray stripe represent the same quantity as those of Figure S1. [6]

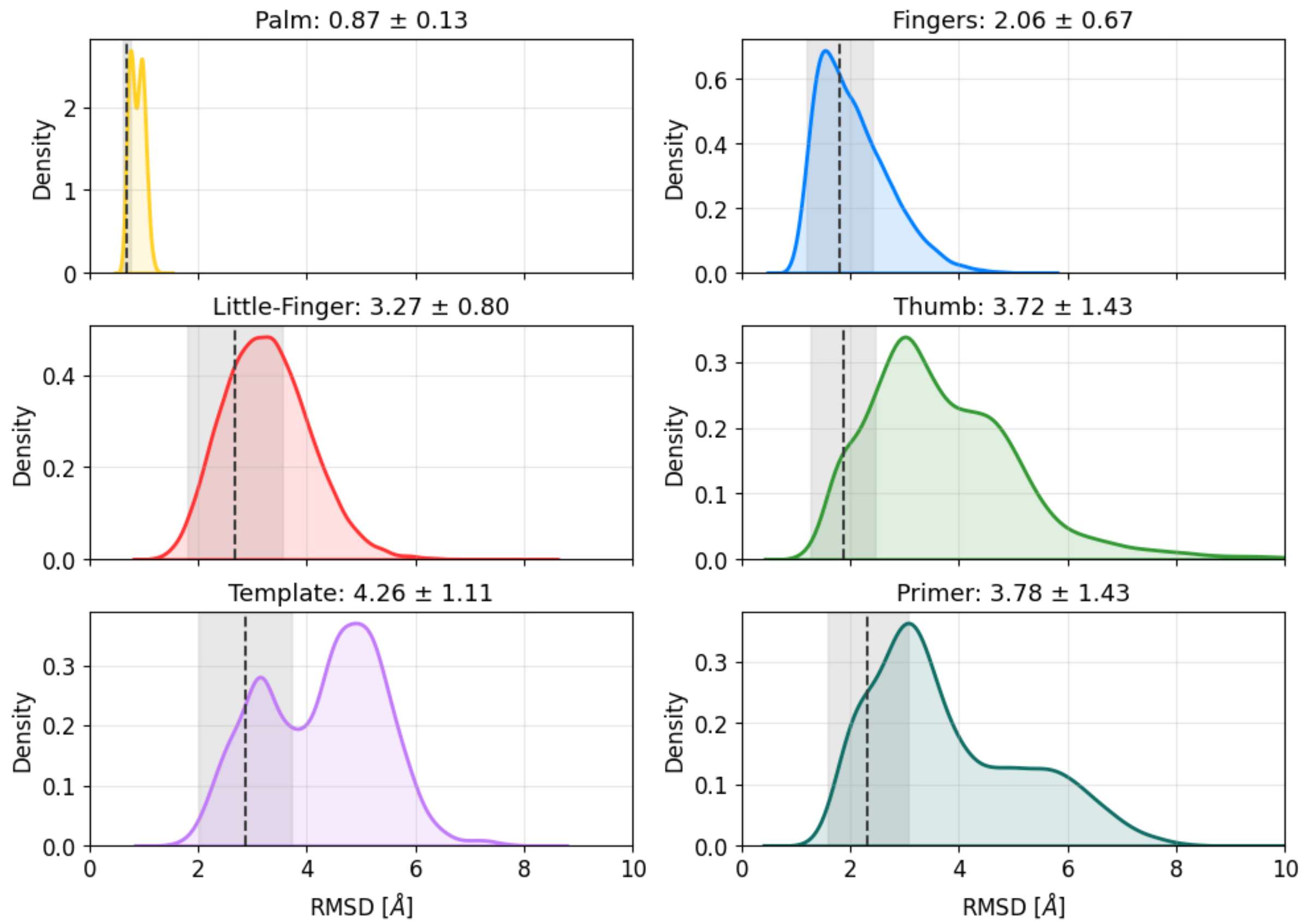

Figure S8.

Root-Mean-Square Deviation (RMSD) of the Gly295Arg variant in state III. (top) Each plot referred to the Palm (gold), Finger (blue), Thumb (green), LF (red), Template (pink), and Primer (teal). The black dashed line and the gray stripe represent the same quantity as those of Figure S1.[6]

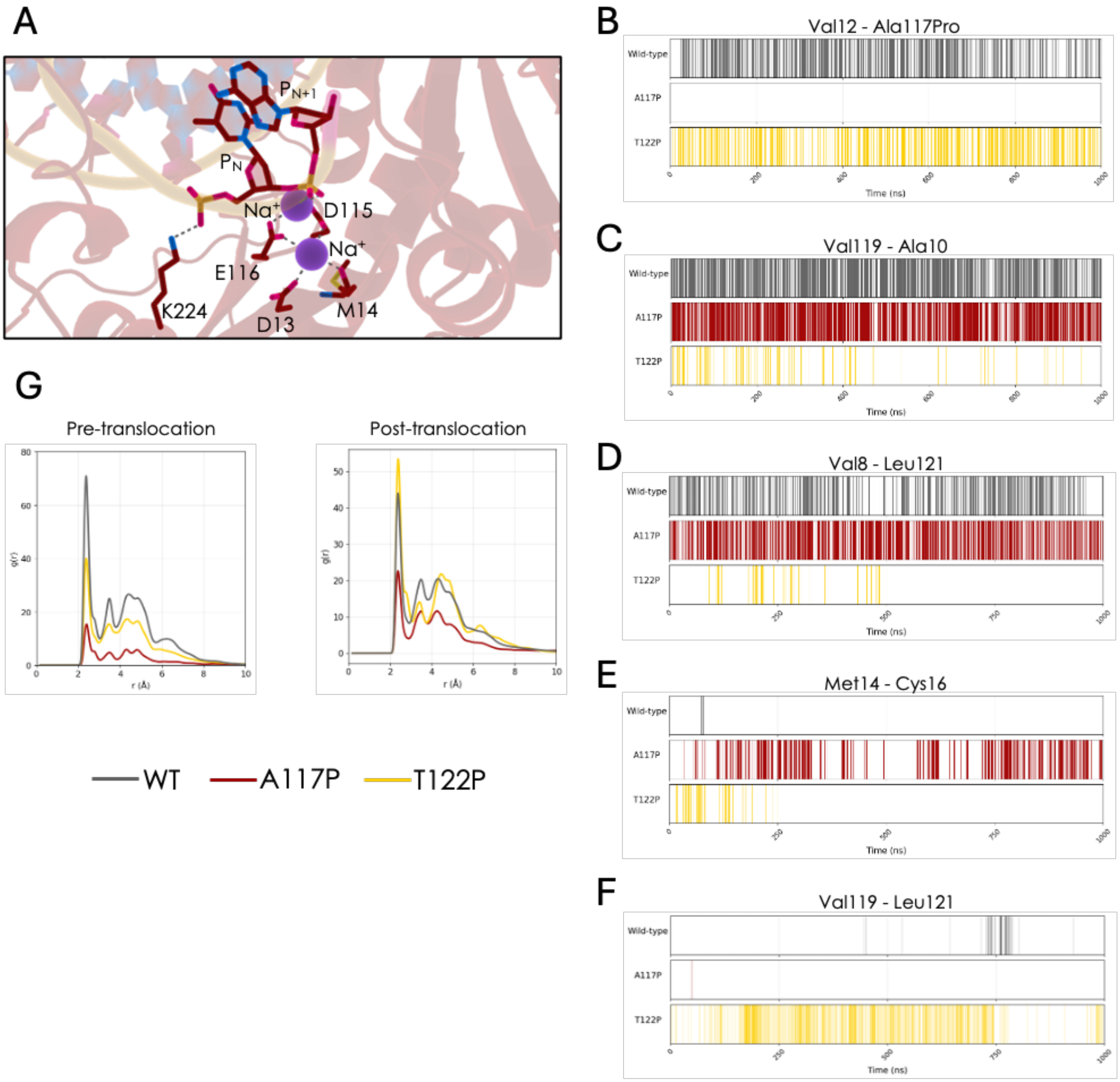

Figure S9.

(A) Reorganization of the catalytic site observed in the Ala117Pro variant. The key residues are shown in stick and colored by atom type. The Na^+ ions are depicted in purple spheres. (from B to F) Heatmap showing the presence of a specific hydrogen bond in the active site. When present (distance < 3 Å), the corresponding time is colored in grey (WT), red (Ala117Pro), or yellow (Thr122Pro). (G) Radial distribution function g(r) calculated for Na^+ ions around 10 Å from the center of mass of the active site (Asp13, Met14, Asp115, and Glu116). Each plot referred to the pre-translocation (left) and post-translocation (right) states, respectively.

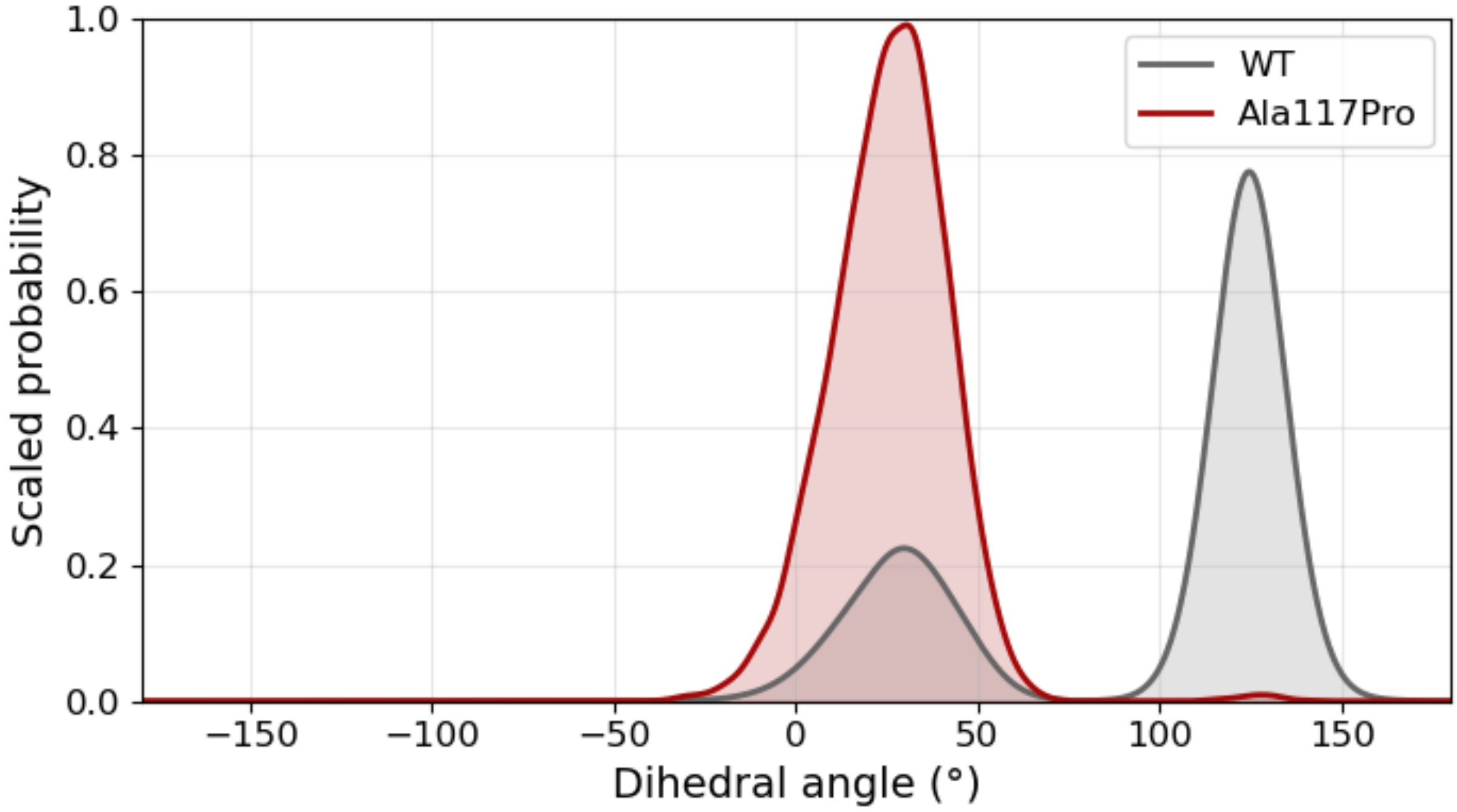

Figure S10.

Probability P_n of dihedral flipping of Asp15 sidechain in the Ala117Pro variant (dark red), compared to WT (grey) in pre-translocation state.[6] The pick around 25° represents the basin when Asp15 is flipped outside the active site, while the pick around 125° represents the basin when Asp15 is straight.[7]

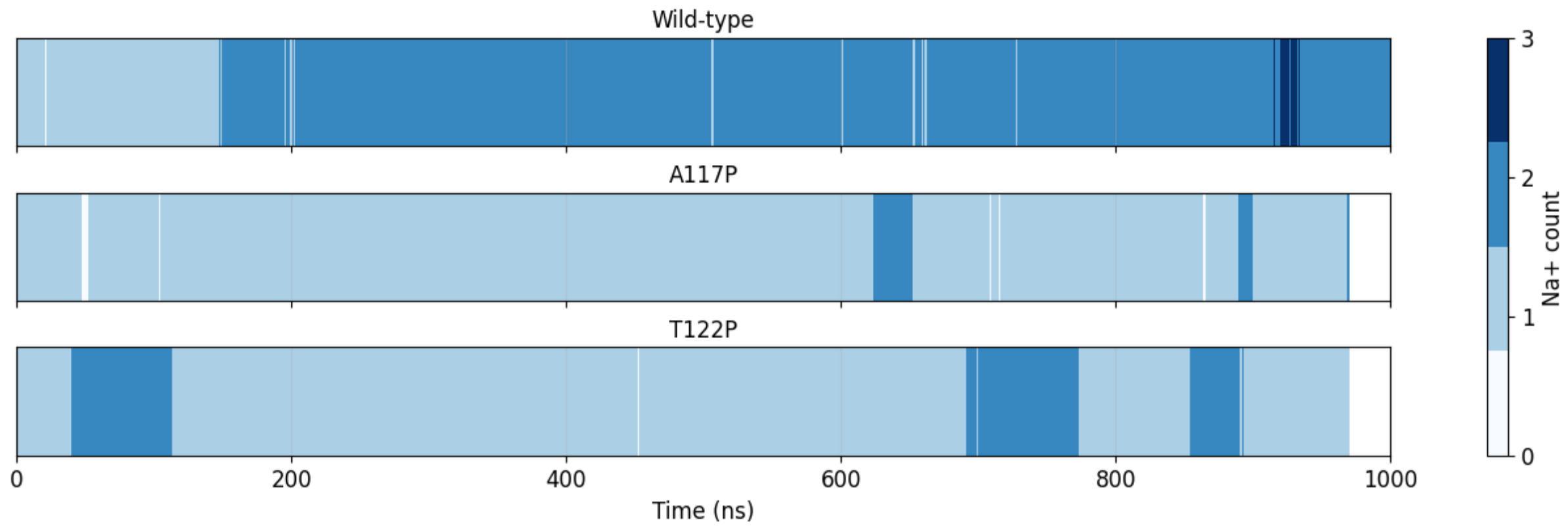

Figure S11.

Heatmap representing the number of Na^+ ions present in the active site over the simulation time. Each line referred to the wild-type, Ala117Pro, and Thr122Pro pre-translocation systems, respectively. The colorbar indicates the number of Na^+ using different shades of blue.[8]

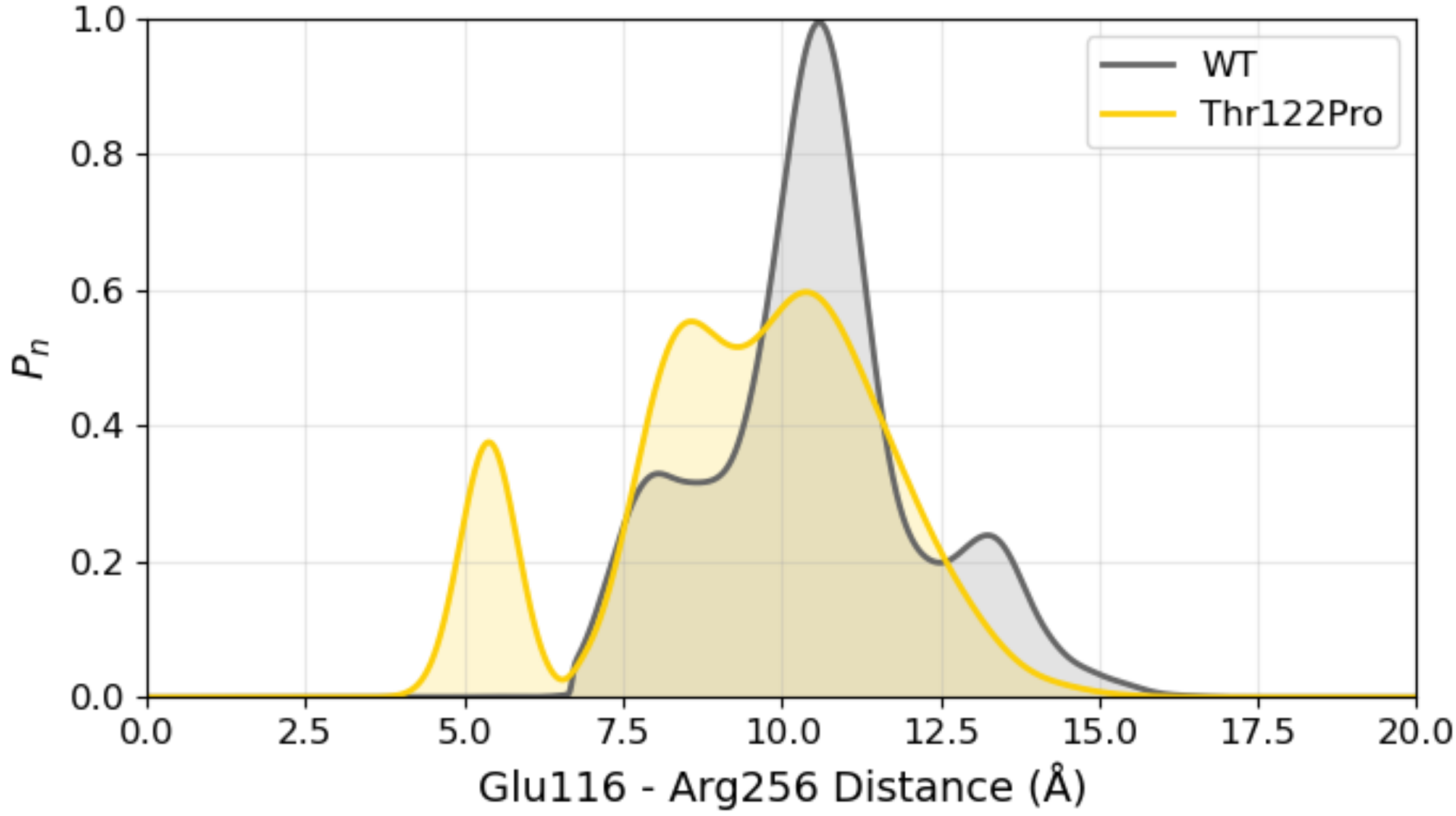

Figure S12.

Probability P_n of interaction between Glu116 and Arg256 in the Thr122Pro variant pre-translocation state (gold), compared to the WT (grey). The lower pick indicates a probability of almost 40% to find the Glu116-Arg256 interaction in the Thr122Pro variant simulation. The distance is calculated between the carbon of the carboxylate group of Glu116 and the central carbon of the guanidinium group of Arg256. [7]

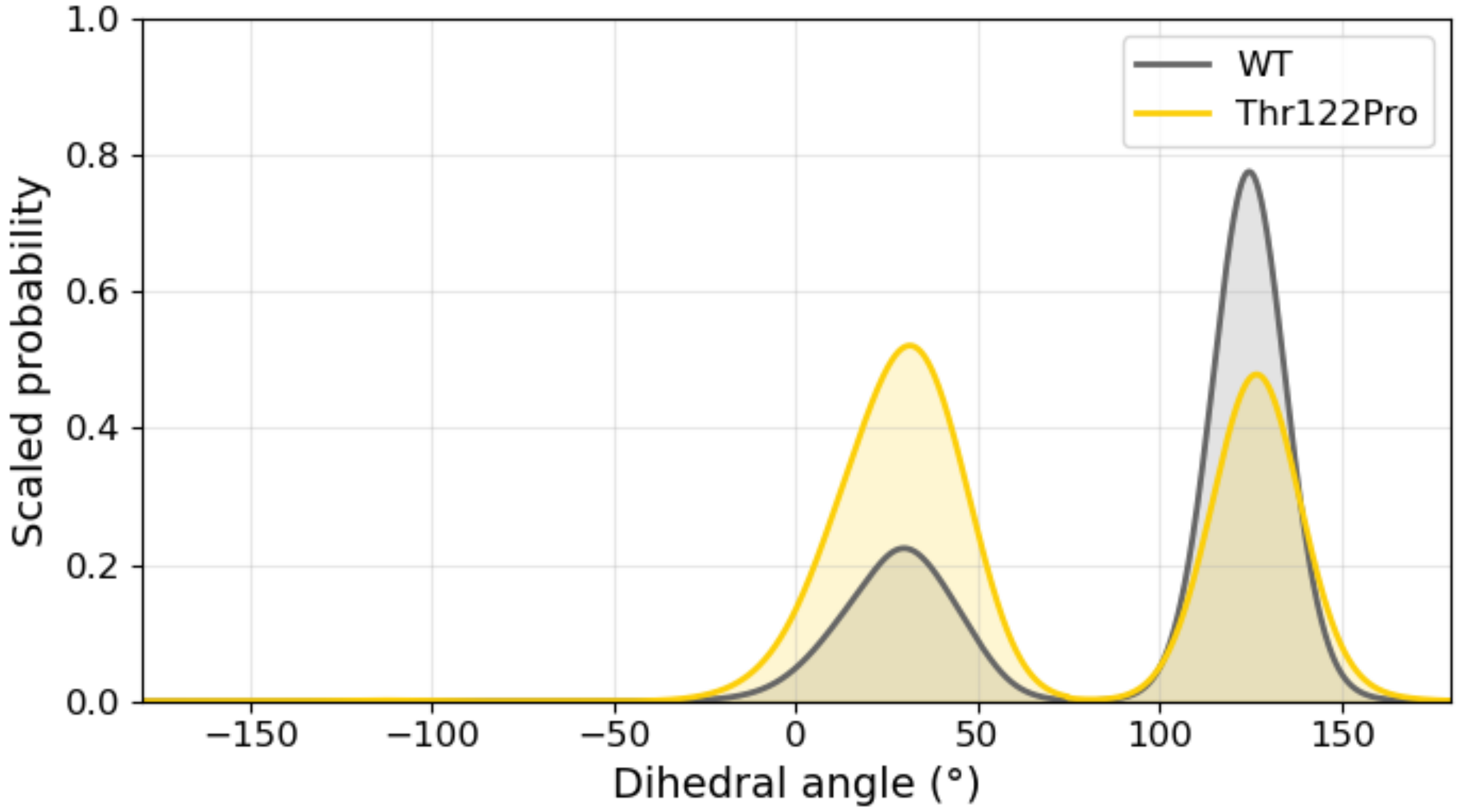

Figure S13.

Probability P_n of dihedral flipping of Asp15 sidechain in the Thr122Pro variant (dark red), compared to WT (grey) in pre-translocation state. The pick around 25° represents the basin when Asp15 is flipped outside the active site, while the pick around 125° represents the basin when Asp15 is straight. [7]

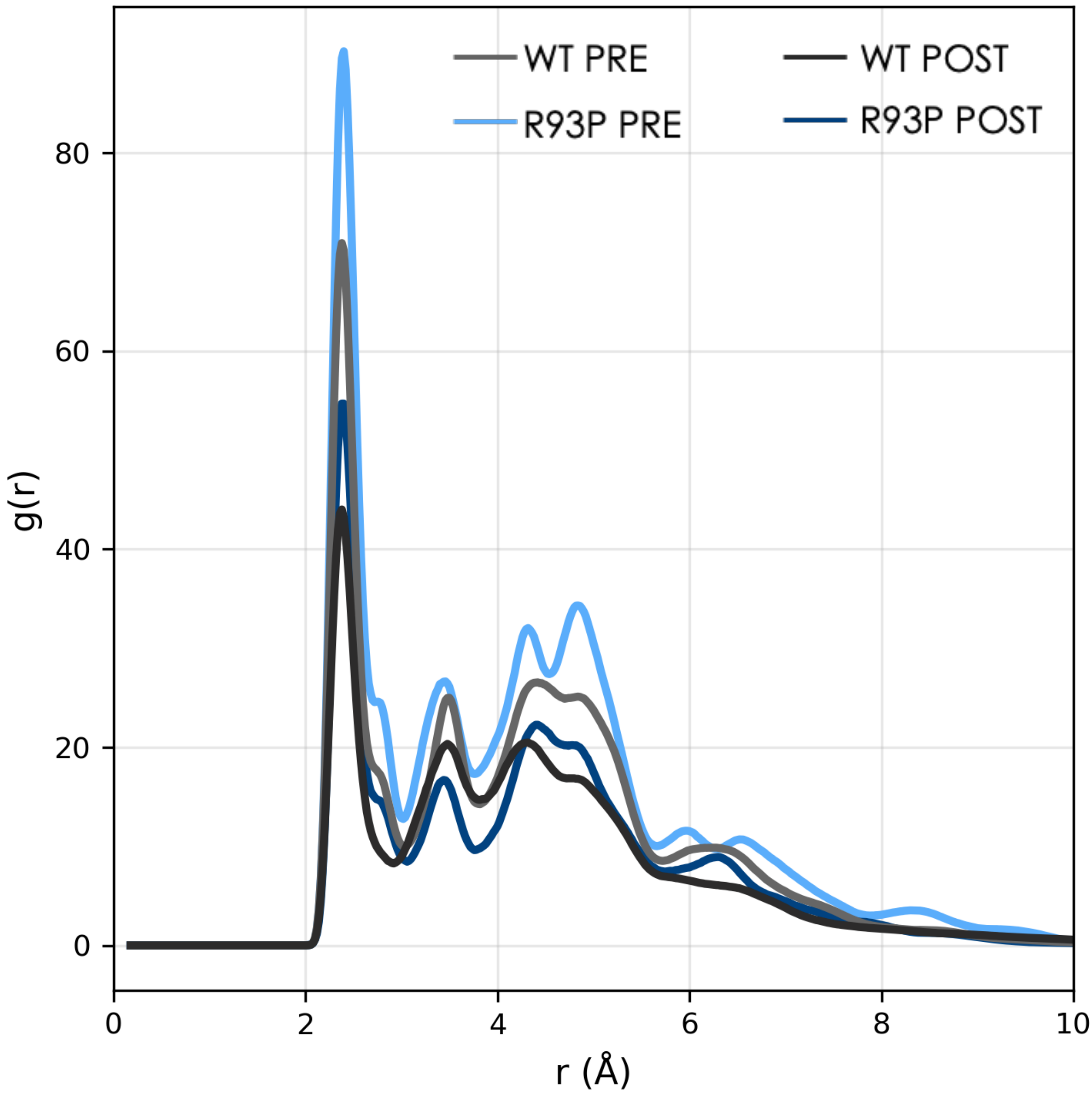

Figure S14.

Radial distribution function g(r) calculated for Na^+ ions around 10 Å from the center of mass of the active site (Asp13, Met14, Asp115, and Glu116) in Arg93Pro, suggesting that the mutation has no impact on the ability of the system to coordinate ions in the active site.[9]

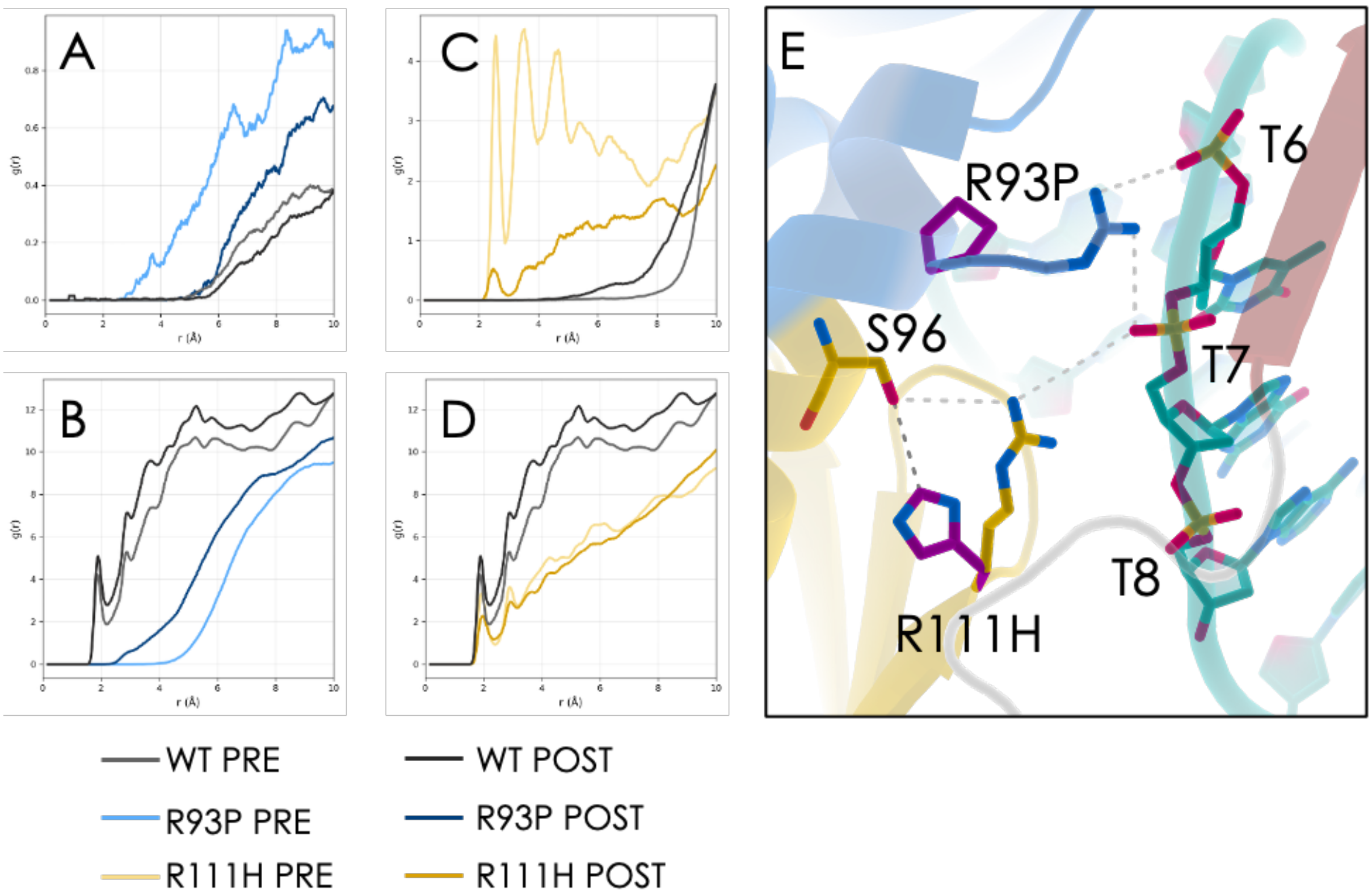

Figure S15.

(left) A-D Radial distribution function g(r) calculated for (A) Na^+ ions around 10 Å from the residue 93; (B) DNA strand around 10 Å from residue 93; (C) Na^+ ions around 10 Å from the residue 111; (D) DNA strand around 10 Å from residue 111. Each plot displays the pre-translocation and post-translocation state data for both WT and mutated systems. (E) The structural view highlights the loss of interaction between the protein and template in the presence of the Arg93Pro and Arg111His mutations (purple stick), compared to the WT (colored by subdomain). [6]

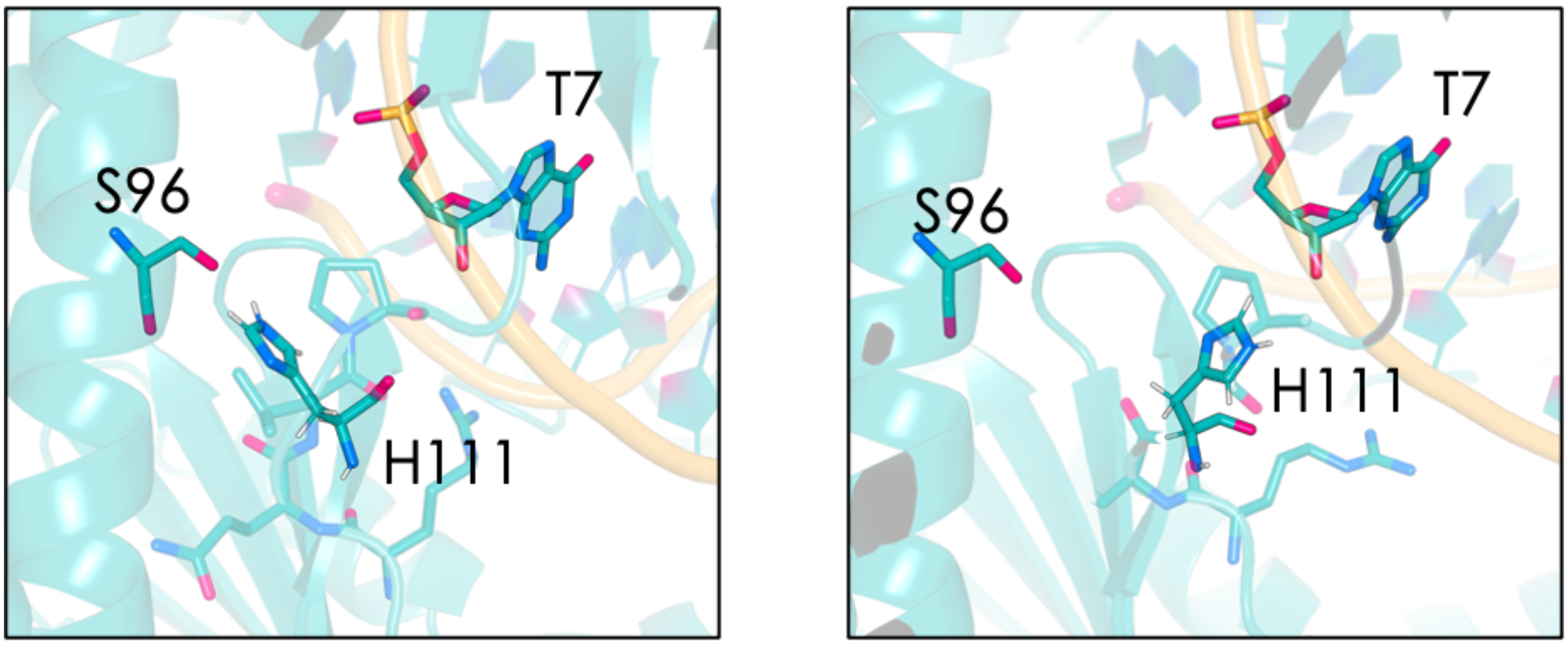

Figure S16.

Close view of His111 in Arg111His variant, highlighting two different conformations of His111 side chain. (left) His111 is interacting with Ser96. (right) His111 is interacting with DNA but not with T7, unlike in the wild-type system. The shorter side chain of His111 does not allow it to interact with phosphate T7, but instead allows it to interact with T6.[6]

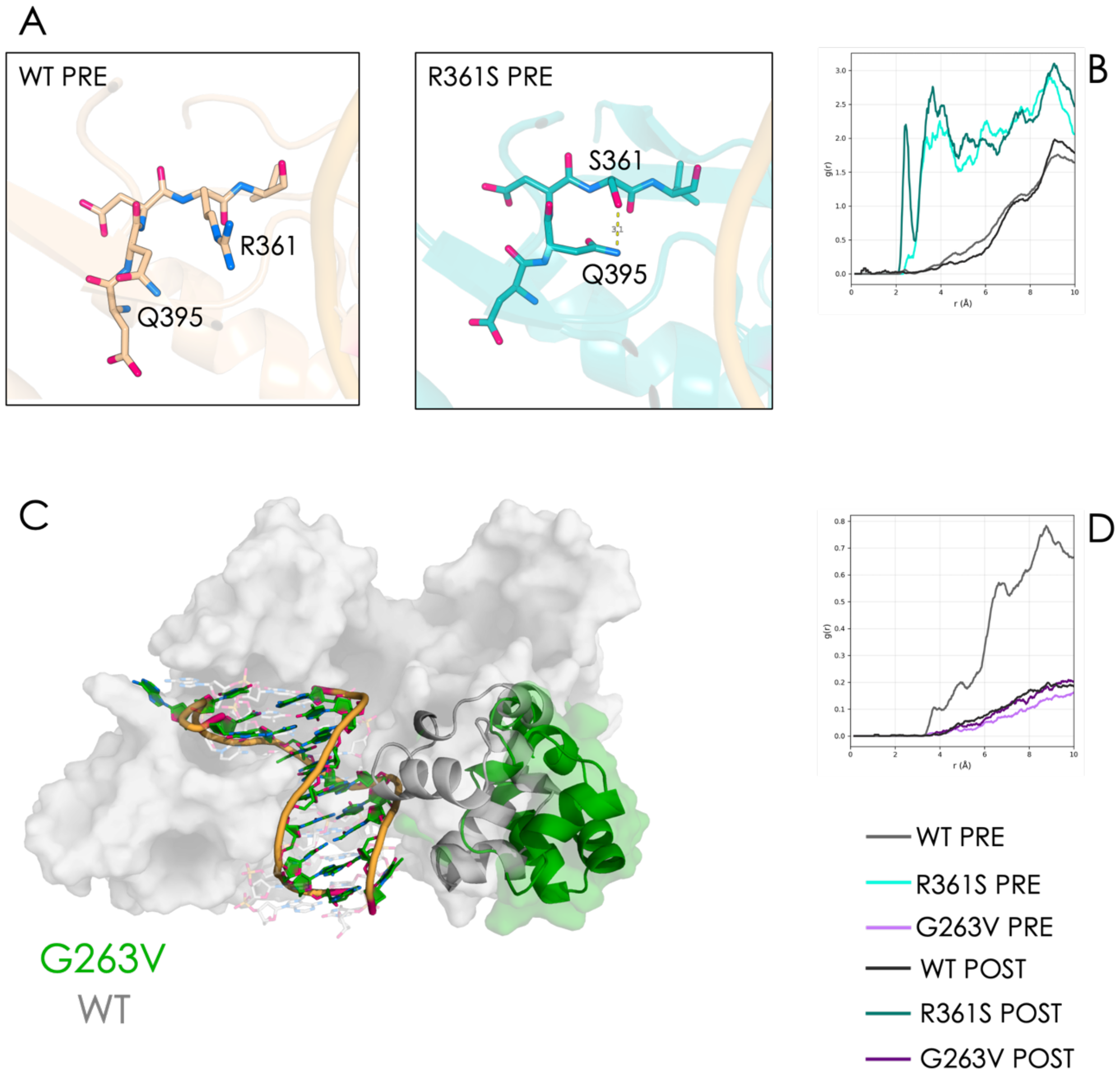

Figure S17.

(A) Structure representation of Polη•DNA system, highlighting the thumb domain detached in Gly263Val variant (green) with respect to the WT system (grey). The protein in the Gly263Val variant is depicted in white surface. The DNA duplex is depicted in white sticks for the WT system and in green cartoons for the Gly263Val variant, to highlight the DNA misalignment. The thumb domain is shown in a cartoon. (B) Radial distribution function g(r) calculated for DNA around 10 Å from the residue 263; (C) Close view in the vicinity of residue 361 in the WT system (left) and in Arg361Ser variant (right). The closed residues are depicted in stick. The protein and the DNA are shown in a cartoon. The newly formed interaction with Asn395 is shown in a yellow dash. (D) Radial distribution function g(r) calculated for the DNA strand around 10 Å from residue 93; (C) Na^+ ions around 10 Å from residue 361.[9]

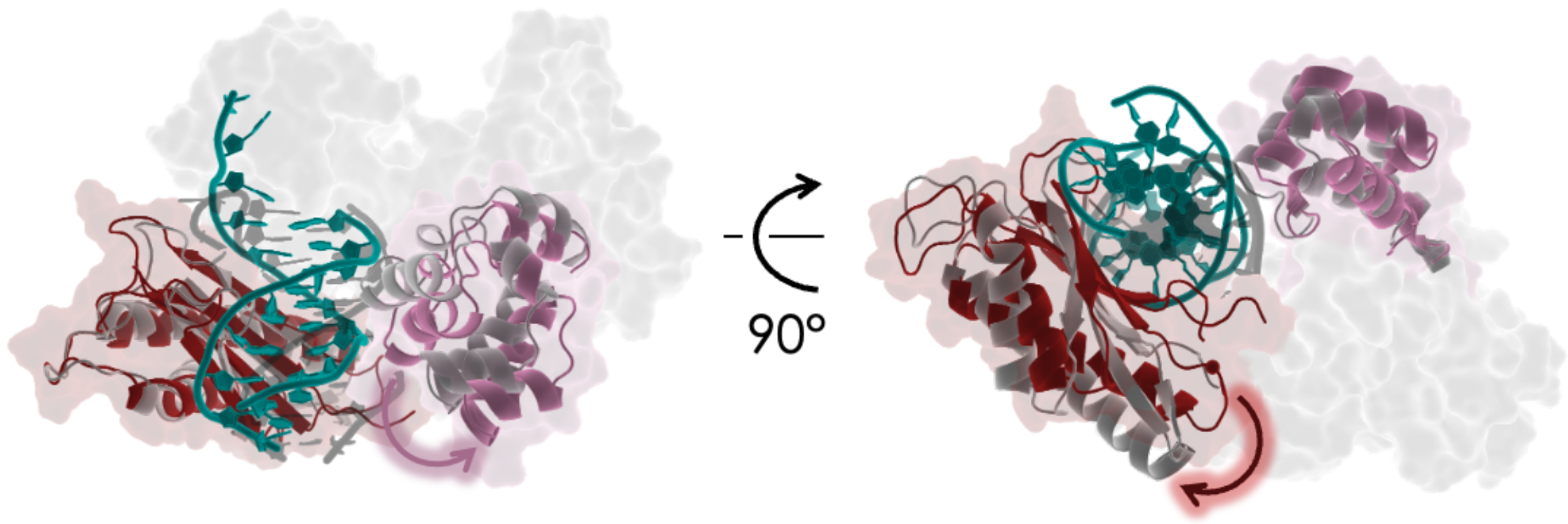

Figure S18.

Structural comparison of the Arg361Ser variant with the WT enzyme in the pre-translocation state.[6] The Arg361Ser protein is shown as a surface representation, with the thumb and LF subdomains highlighted in cartoon (pink and red, respectively). The DNA is shown in cartoon and colored teal for Arg361Ser and grey for the wild type. The left panel shows the system in a front view, with the DNA axis oriented vertically, highlighting the rearrangement of the thumb subdomain. The right panel shows the same structure rotated by 90°, providing a bottom view in which the DNA axis enters the plane of the figure. From this angle, the closure of the large helix in the LF subdomain becomes clearly visible. Together, the motions of these two subdomains result in a misalignment of the DNA, as also evident in the left panel.

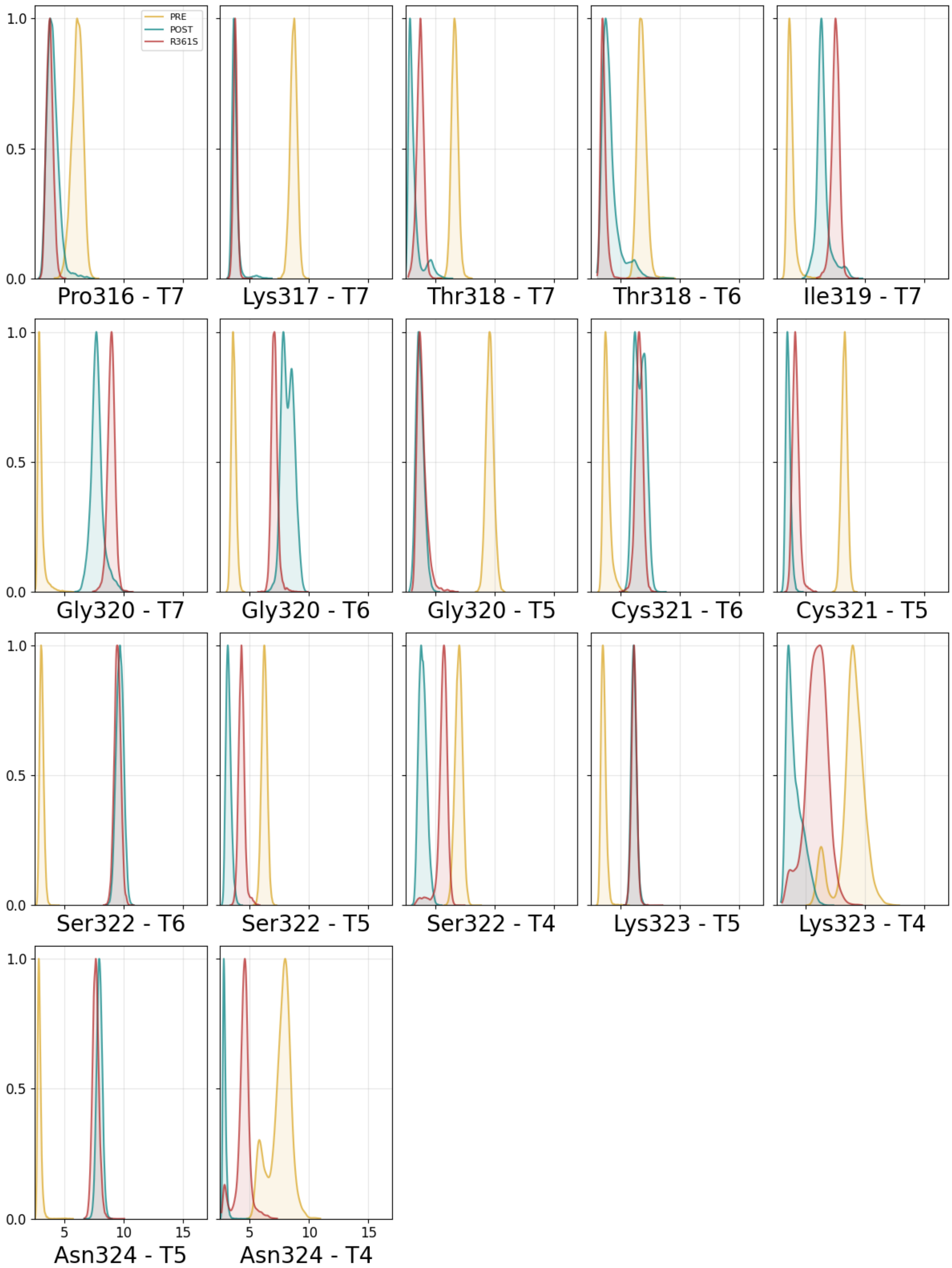

Figure S19.

Probability distributions of molecular splint residue-DNA distances in the Arg361Ser pre-translocation state variant. Distances of wild-type pre-translocation (gold), post-translocation (teal), and Arg361Ser variant (teal) are shown.[6] All the distances are calculated with respect to the phosphate atom of the DNA substrate.

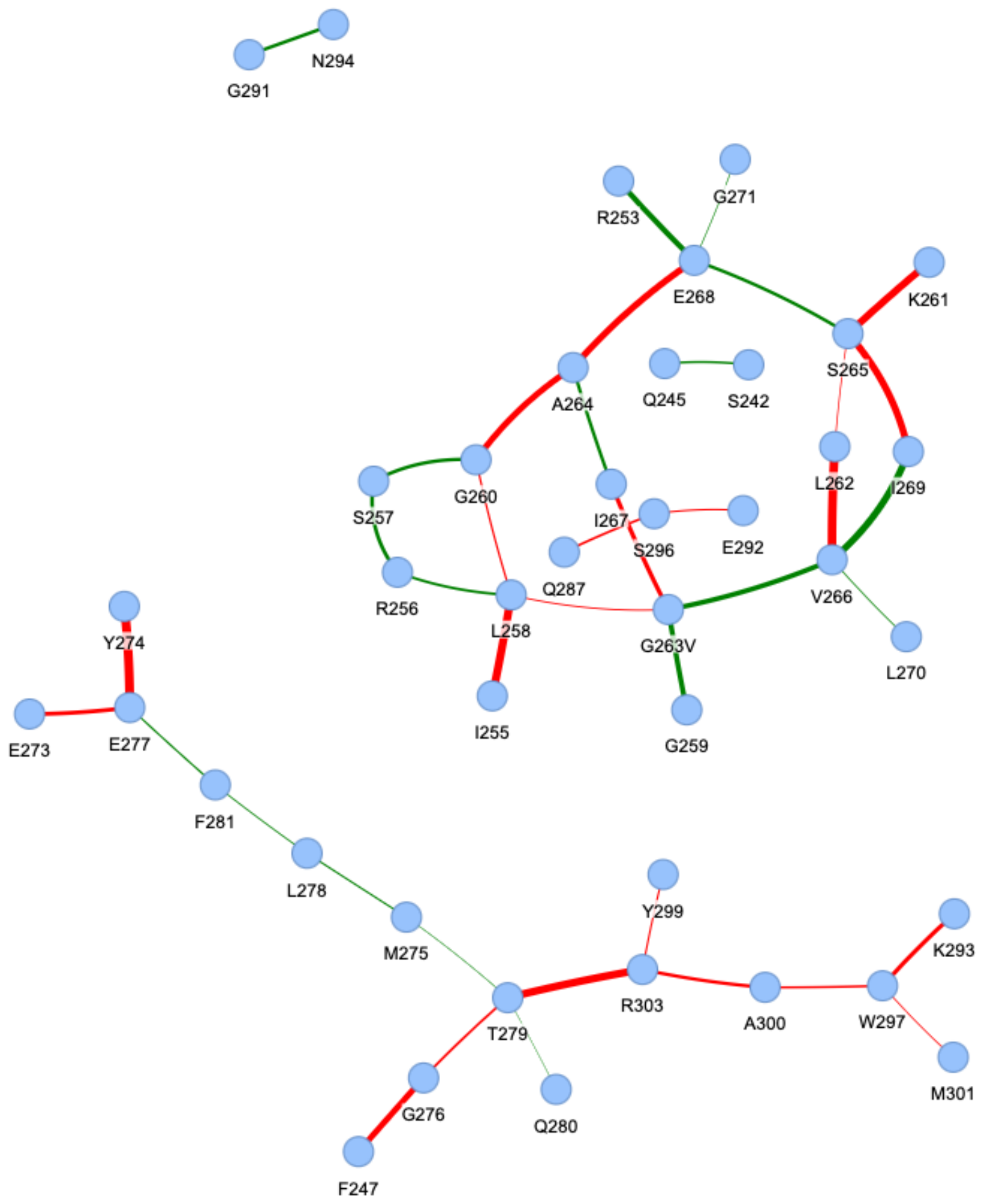

Figure S20.

Hydrogen-bond network in the Gly263Val variant within the thumb subdomain backbone (residues 240–305), calculated using Bridge2.[10,11] Each involved residue is represented as a node in the graph, while edges indicate hydrogen bonds between residue pairs. Only interactions whose occupancy differs from the wild type by more than 10% are shown. Colors indicate whether the occupancy decreases (red) or increases (green) in the Gly263Val variant relative to the WT system. Line thickness reflects the magnitude of the occupancy change, with thicker edges corresponding to larger differences.

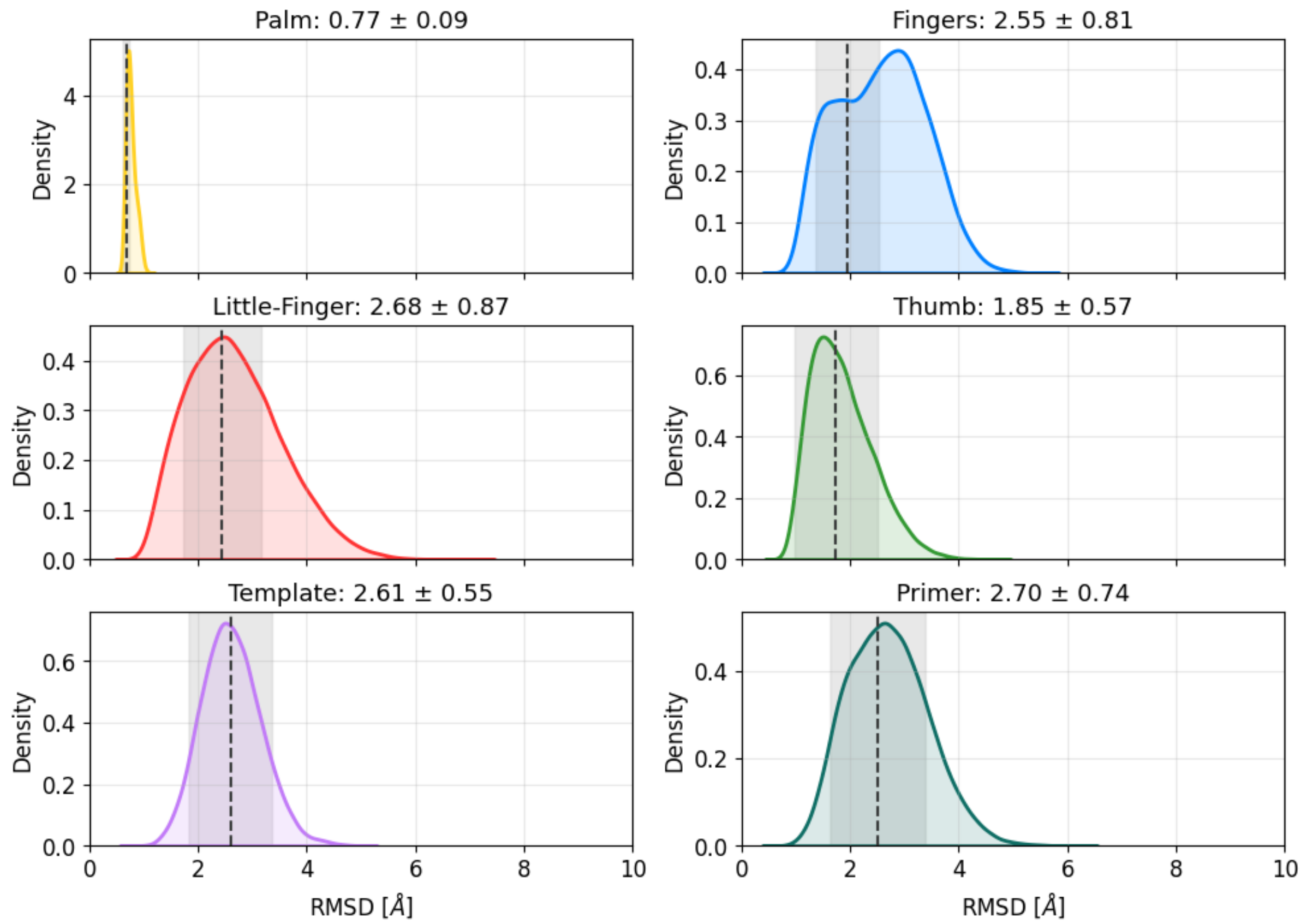

Figure S21.

Root-Mean-Square Deviation (RMSD) of the Ala117Pro variant in state IV. (top) Each plot referred to the Palm (gold), Finger (blue), Thumb (green), LF (red), Template (pink), and Primer (teal). The black dashed line and the gray stripe represent the mean value and the standard deviation in the WT state IV.[6]

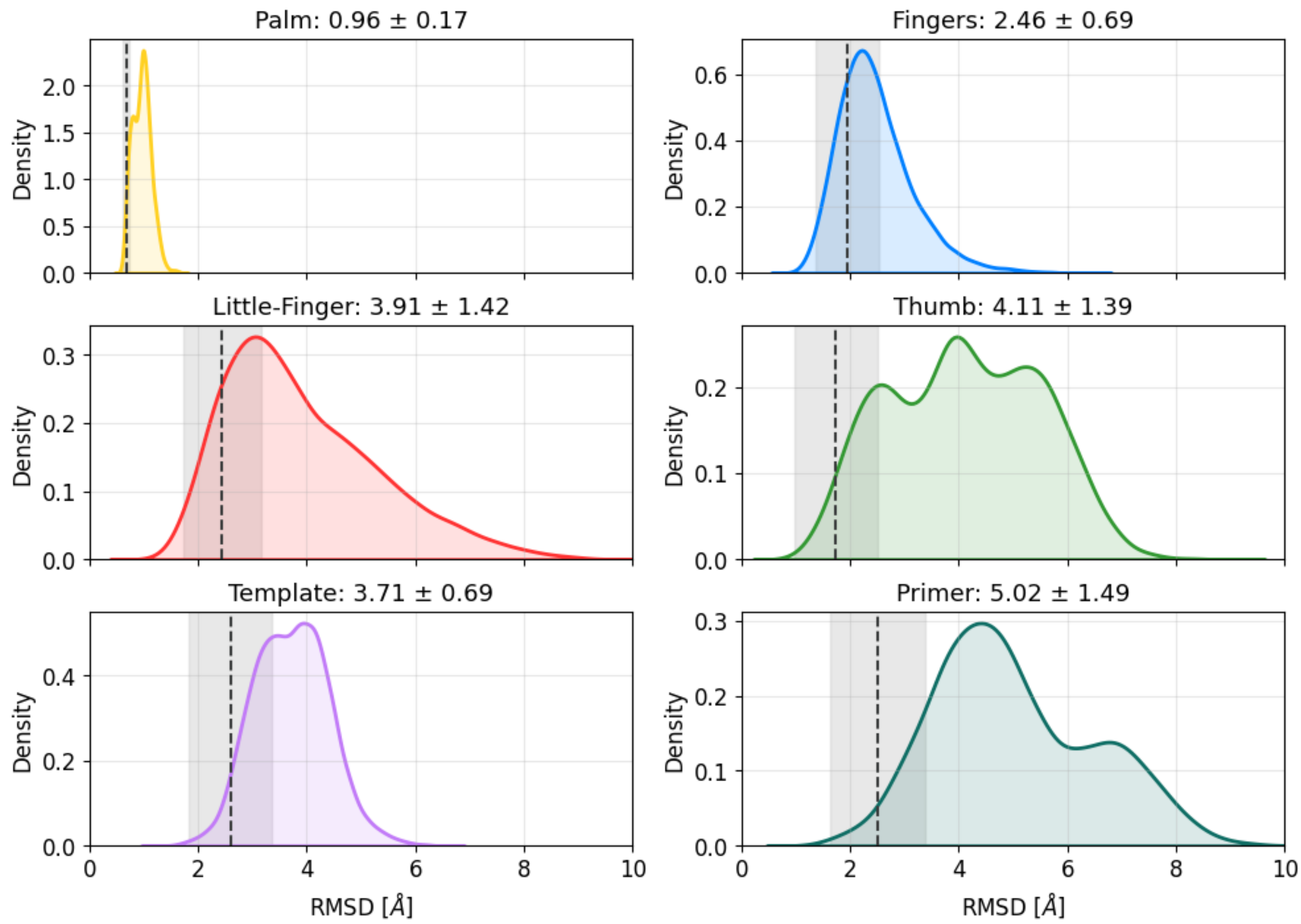

Figure S22.

Root-Mean-Square Deviation (RMSD) of the Thr122Pro variant in state IV. (top) Each plot referred to the Palm (gold), Finger (blue), Thumb (green), LF (red), Template (pink), and Primer (teal). The black dashed line and the gray stripe represent the same quantity as those of Figure S21.[6]

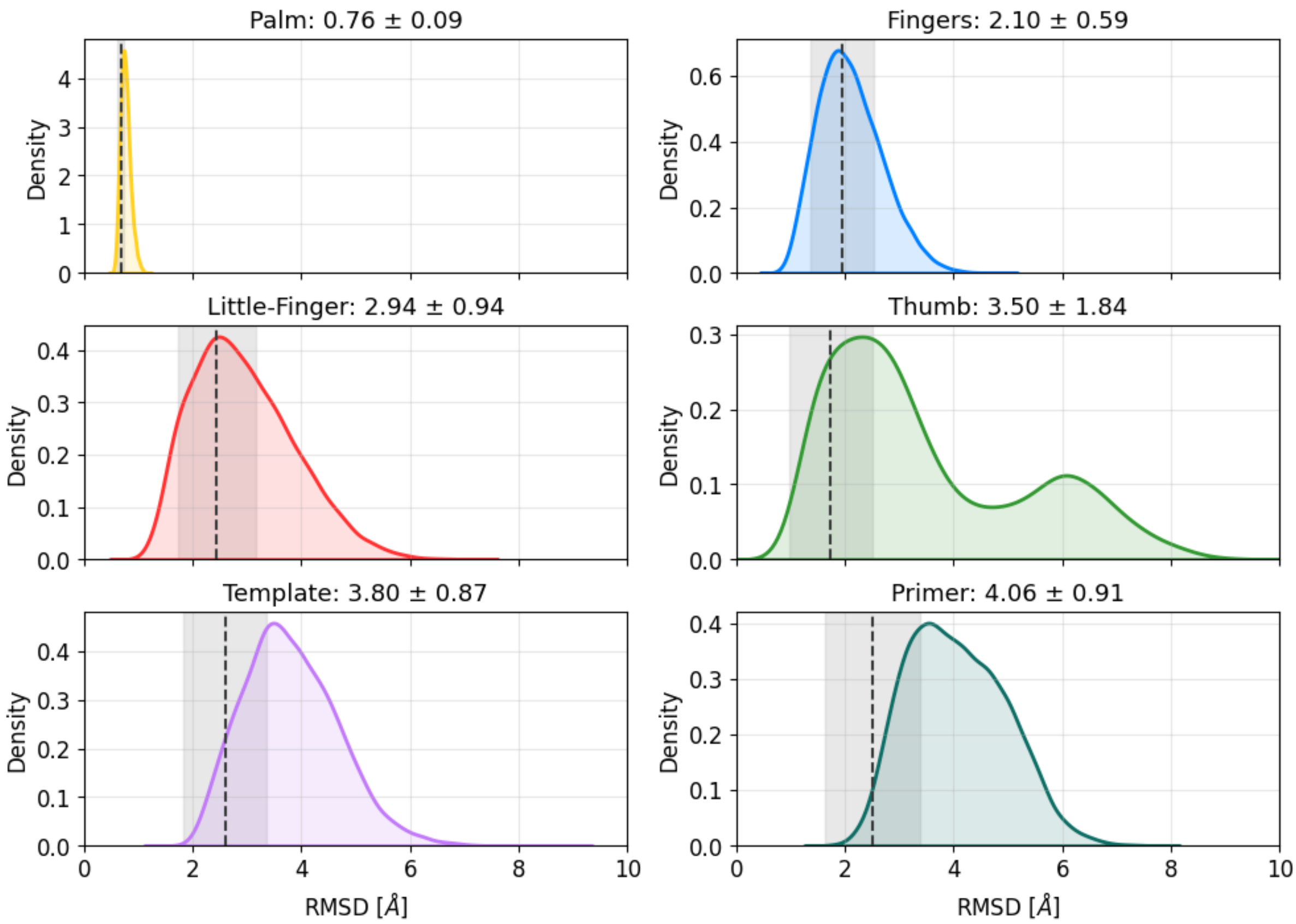

Figure S23.

Root-Mean-Square Deviation (RMSD) of the Arg93Pro variant in state IV. (top) Each plot referred to the Palm (gold), Finger (blue), Thumb (green), LF (red), Template (pink), and Primer (teal). The black dashed line and the gray stripe represent the same quantity as those of Figure S21.[6]

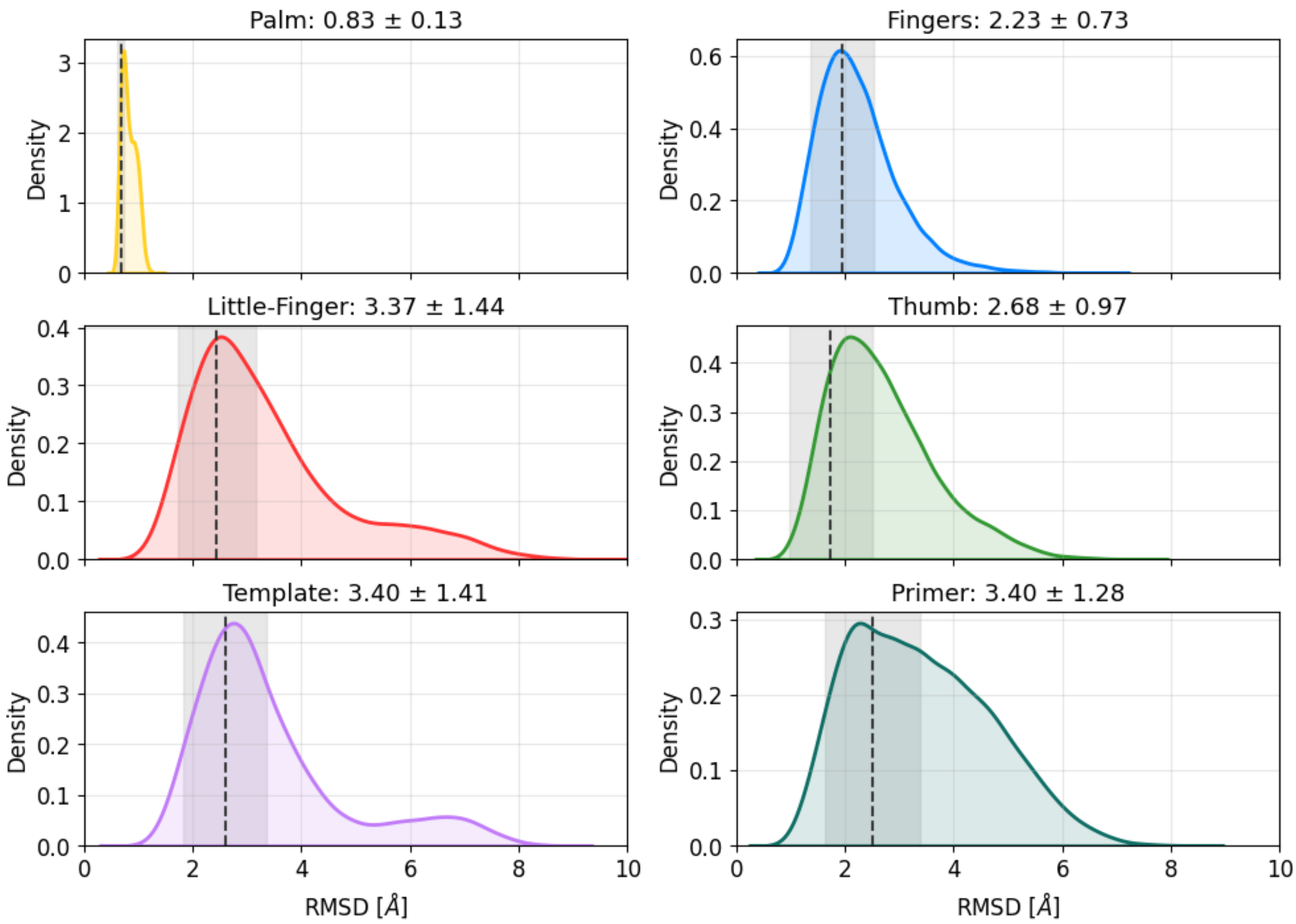

Figure S24.

Root-Mean-Square Deviation (RMSD) of the Arg111His variant in state IV. (top) Each plot referred to the Palm (gold), Finger (blue), Thumb (green), LF (red), Template (pink), and Primer (teal). The black dashed line and the gray stripe represent the same quantity as those of Figure S21.[6]

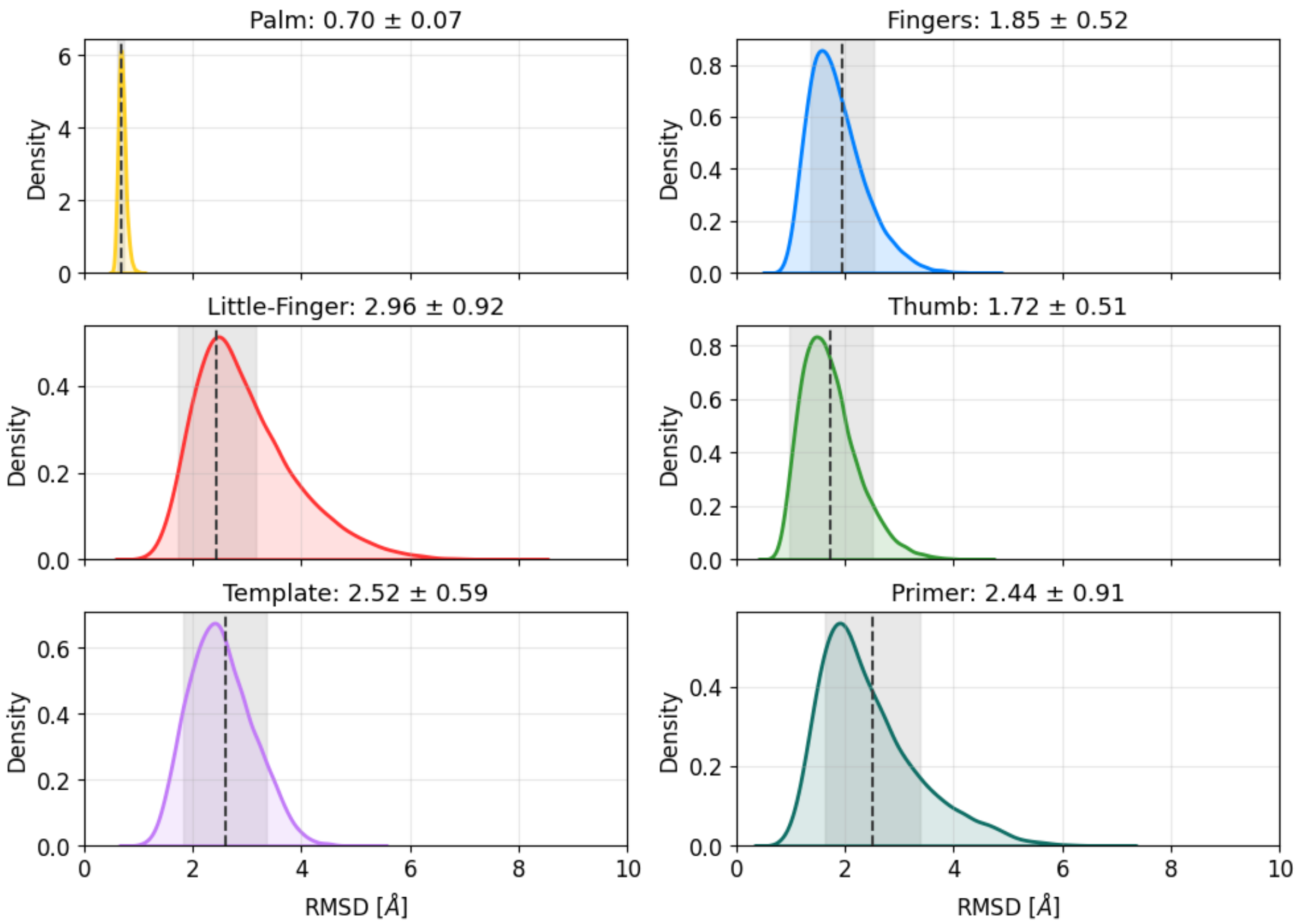

Figure S25.

Root-Mean-Square Deviation (RMSD) of the Arg361Ser variant in state IV. (top) Each plot referred to the Palm (gold), Finger (blue), Thumb (green), LF (red), Template (pink), and Primer (teal). The black dashed line and the gray stripe represent the same quantity as those of Figure S21.[6]

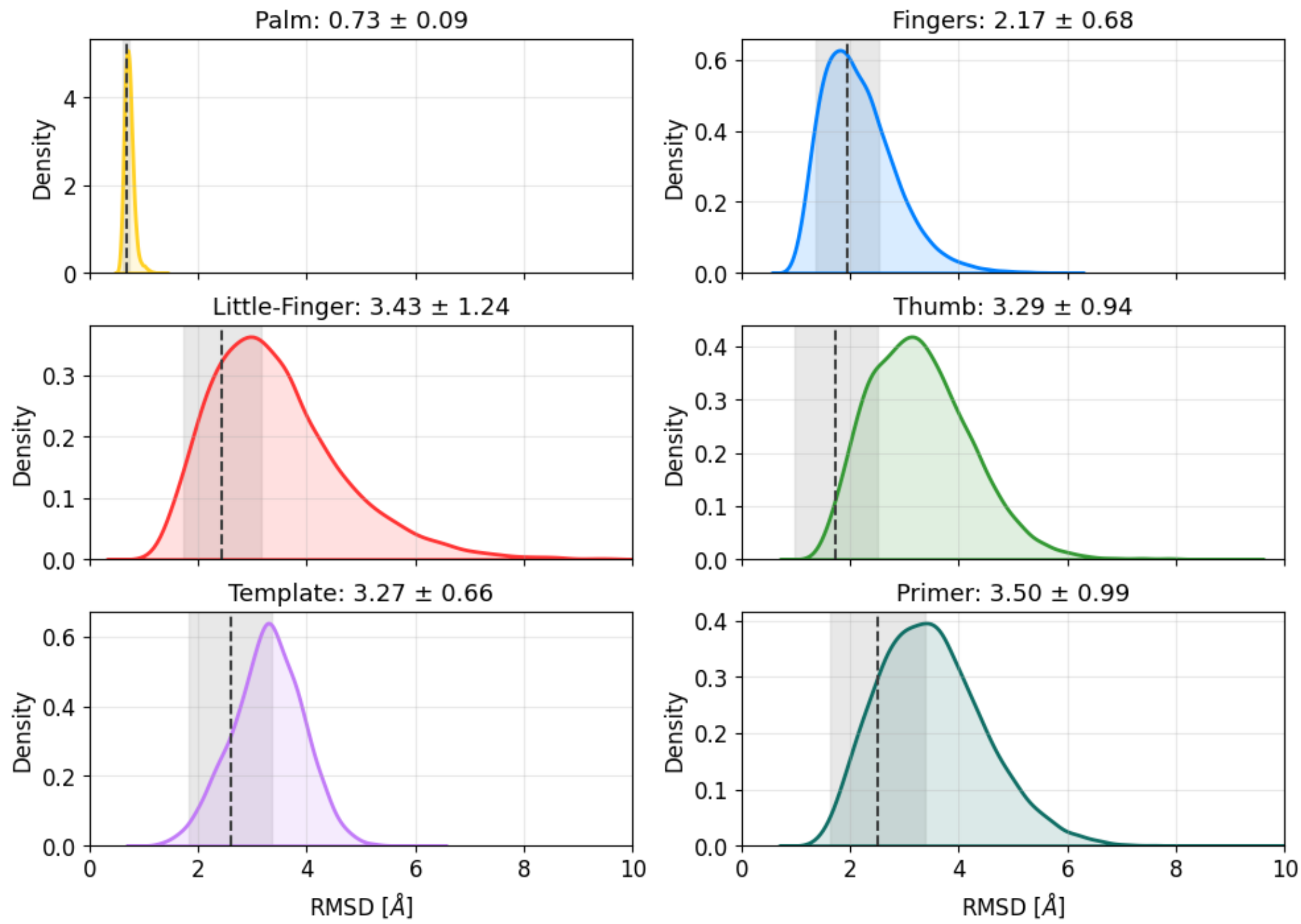

Figure S26.

Root-Mean-Square Deviation (RMSD) of the Gly263Val variant in state IV. (top) Each plot referred to the Palm (gold), Finger (blue), Thumb (green), LF (red), Template (pink), and Primer (teal). The black dashed line and the gray stripe represent the same quantity as those of Figure S21.[6]

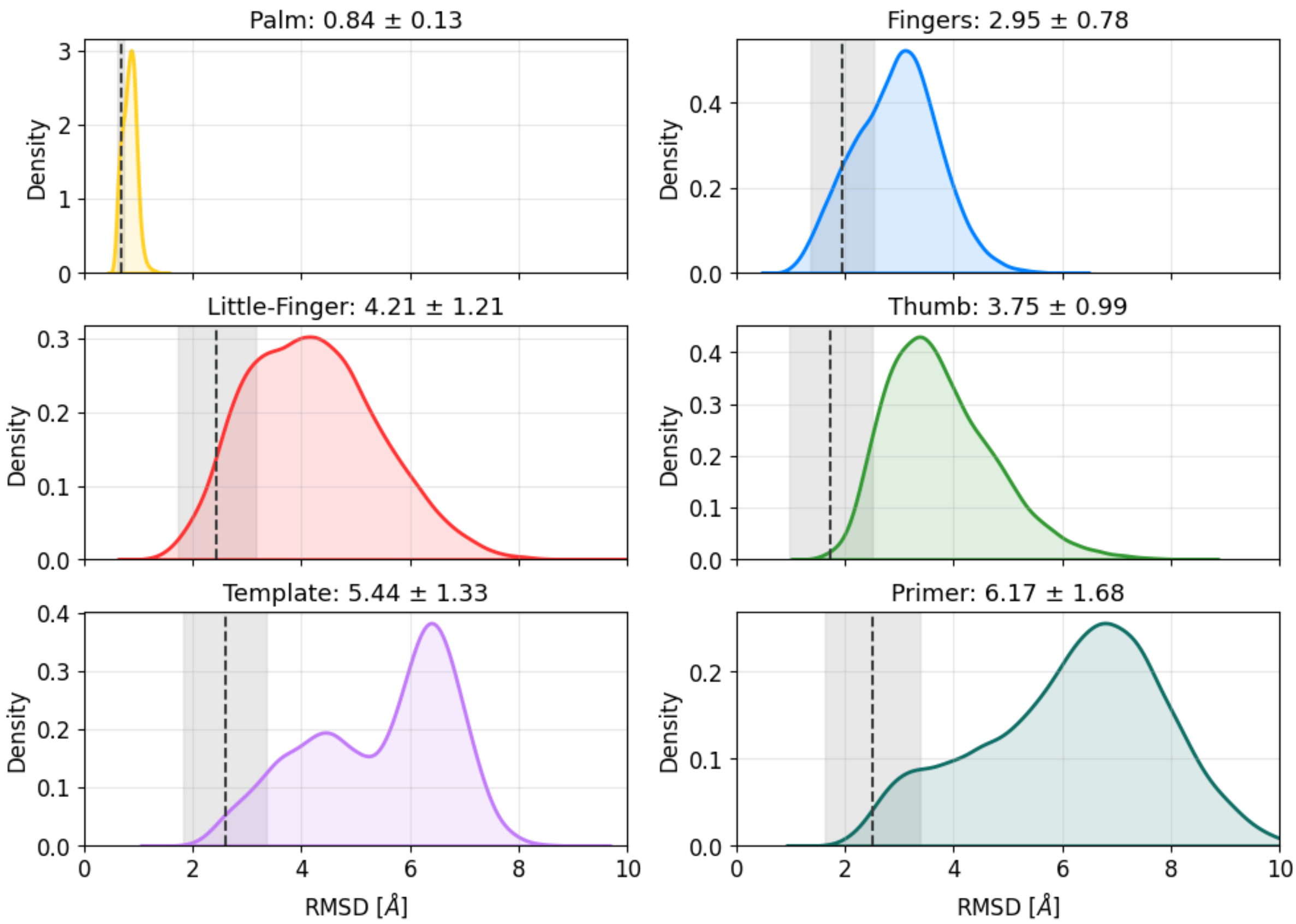

Figure S27.

Root-Mean-Square Deviation (RMSD) of the Val266Asp variant in state IV. (top) Each plot referred to the Palm (gold), Finger (blue), Thumb (green), LF (red), Template (pink), and Primer (teal). The black dashed line and the gray stripe represent the same quantity as those of Figure S21.[6]

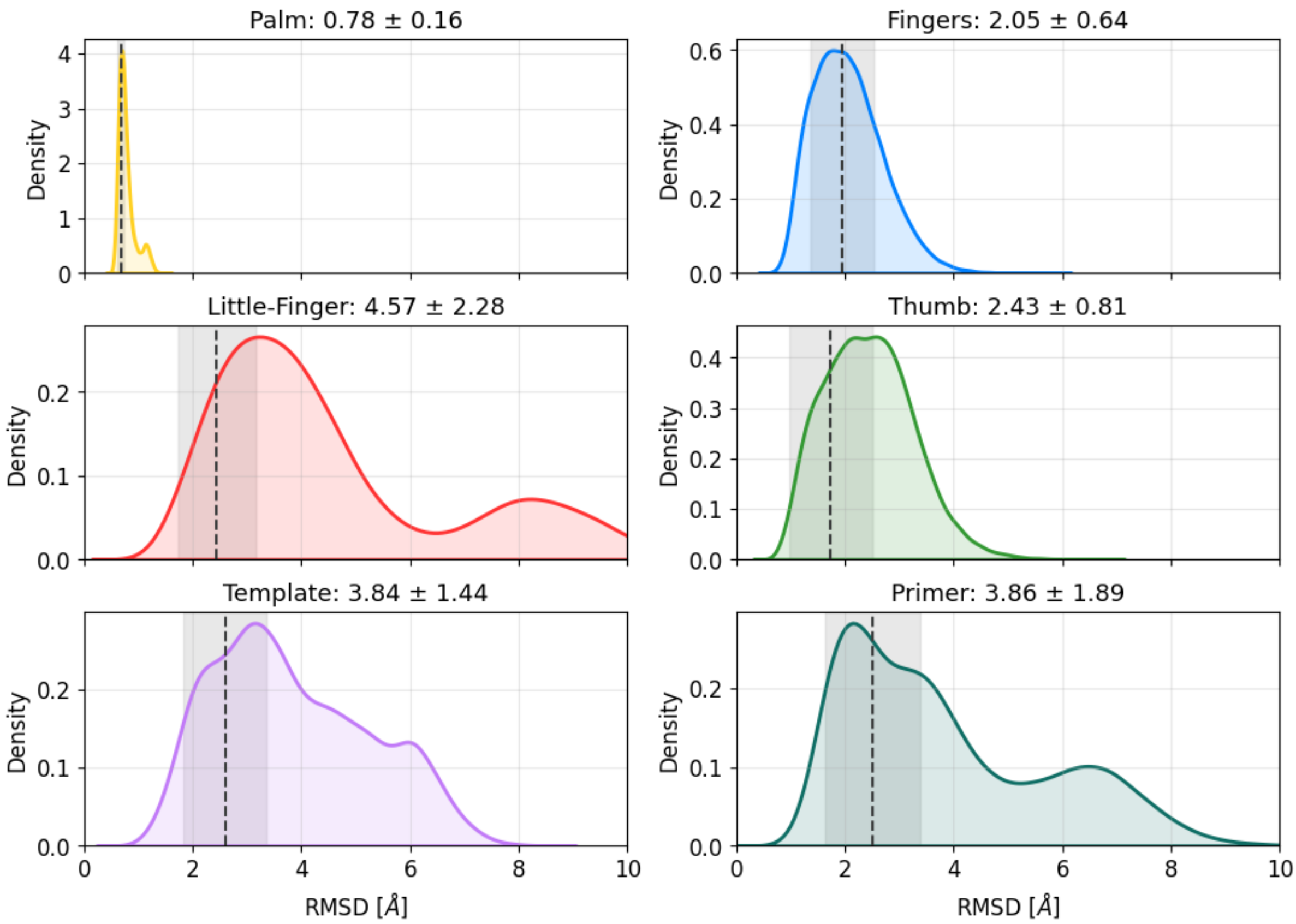

Figure S28.

Root-Mean-Square Deviation (RMSD) of the Gly295Arg variant in state IV. (top) Each plot referred to the Palm (gold), Finger (blue), Thumb (green), LF (red), Template (pink), and Primer (teal). The black dashed line and the gray stripe represent the same quantity as those of Figure S21.[6]

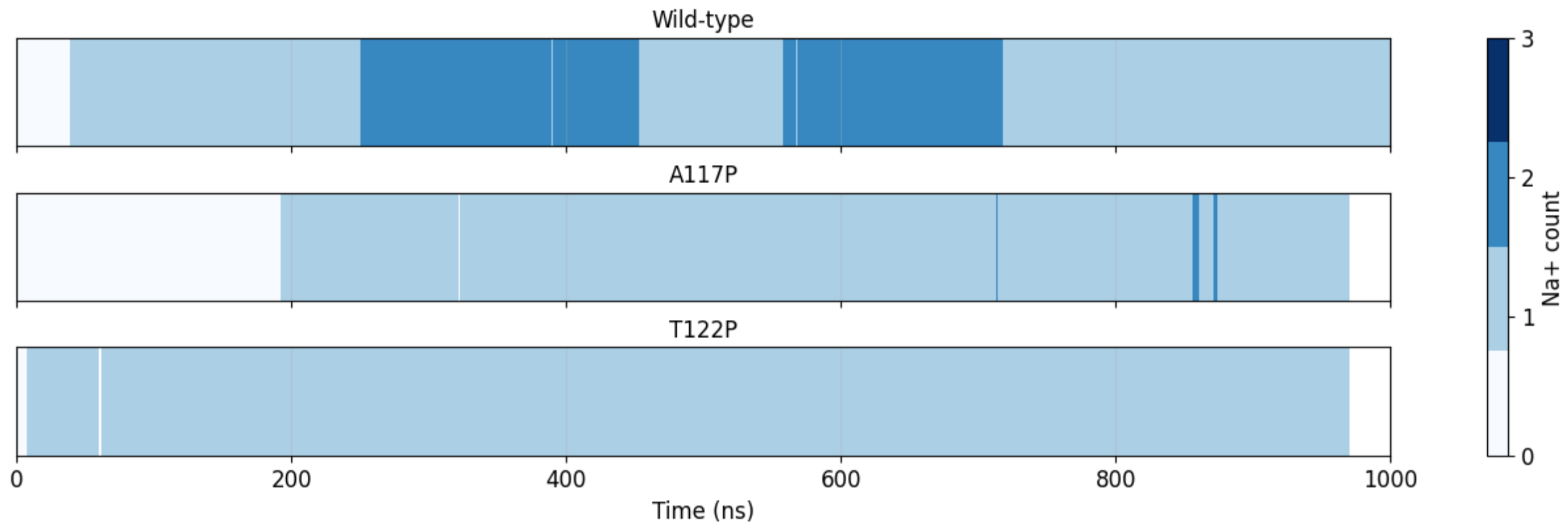

Figure S29.

Heatmap representing the number of Na^+ ions present in the active site over the simulation time. Each line referred to the wild-type, Ala117Pro, and Thr122Pro post-translocation systems, respectively. The colorbar indicates the number of Na^+ using different shades of blue.[8]

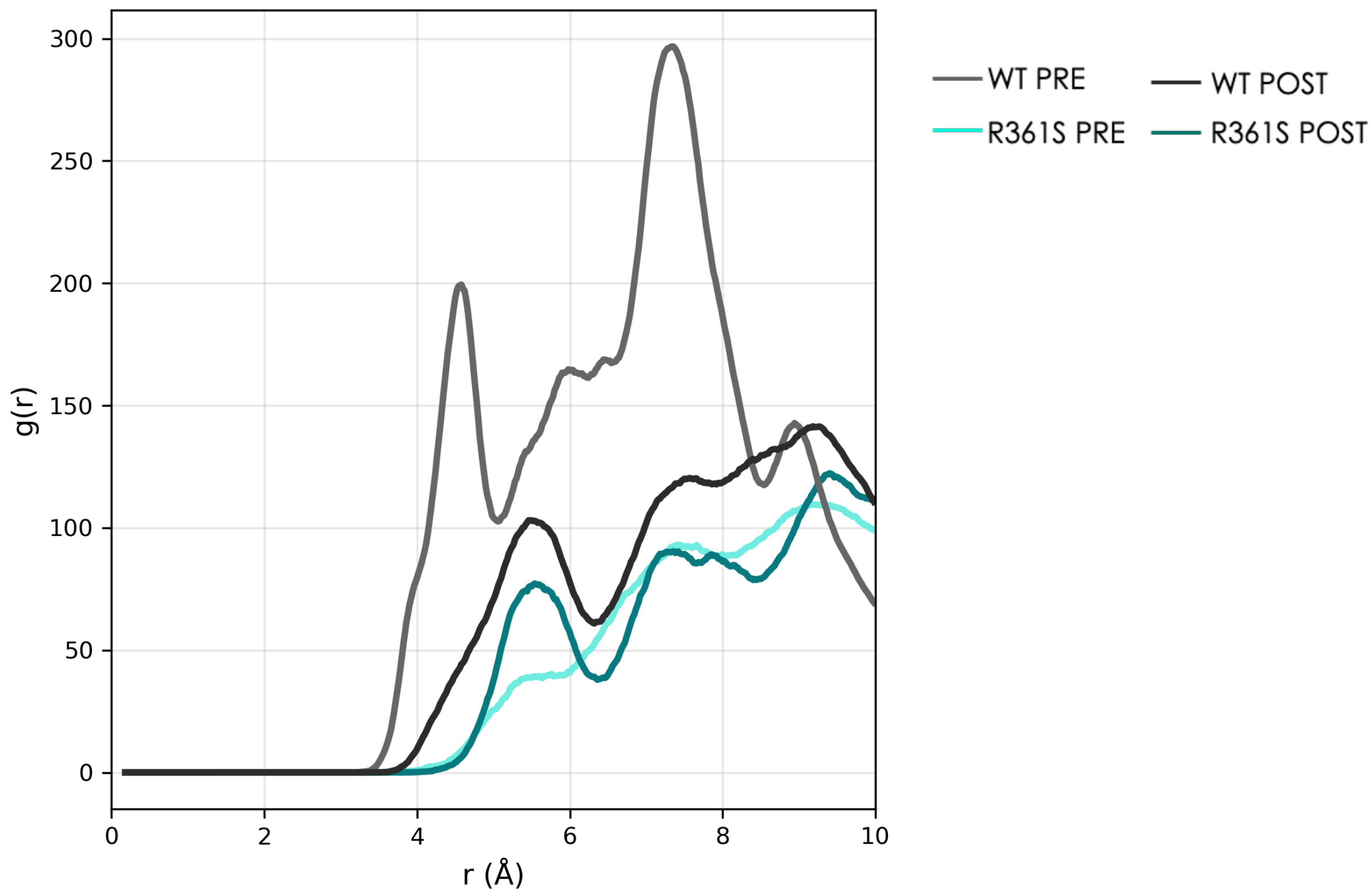

Figure S30.

Radial distribution function g(r) calculated for the phosphate atom P9 (corresponding to the last added nucleotide) around 10 Å from the residue 361 in Arg361Ser and the WT system.[9]

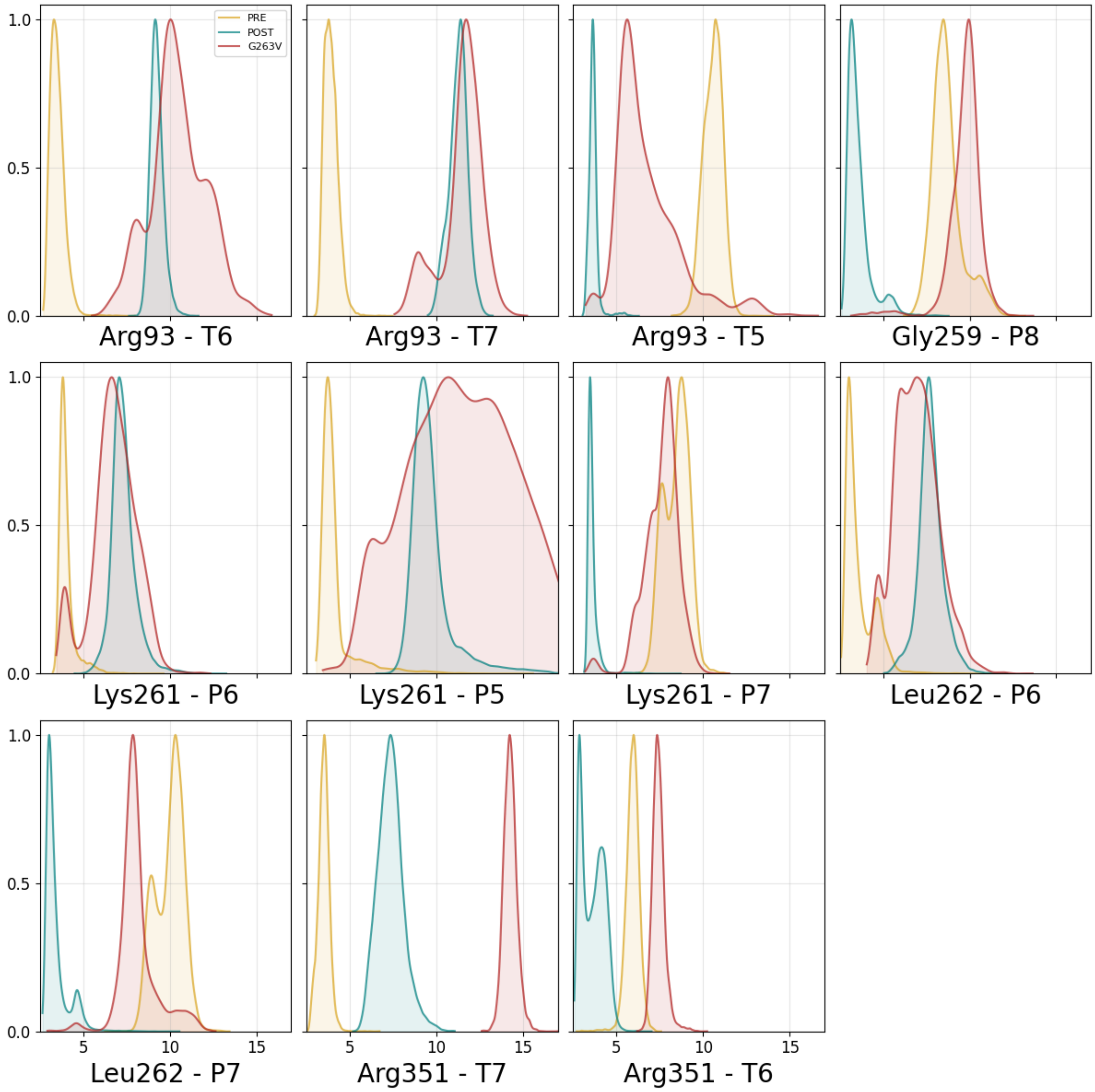

Figure S31.

Probability distributions of key residue–DNA distances in the Gly263Val post-translocation state variant. Distances of wild-type pre-translocation (gold), post-translocation (teal), and Gly263Val variant (teal) are shown. All the distances are calculated with respect to the phosphate atom of the DNA substrate. The distances involving a lysine are calculated using the nitrogen atom of the side chain. The distances involving an arginine are calculated using the carbon atom of the guanidinium group.[6]

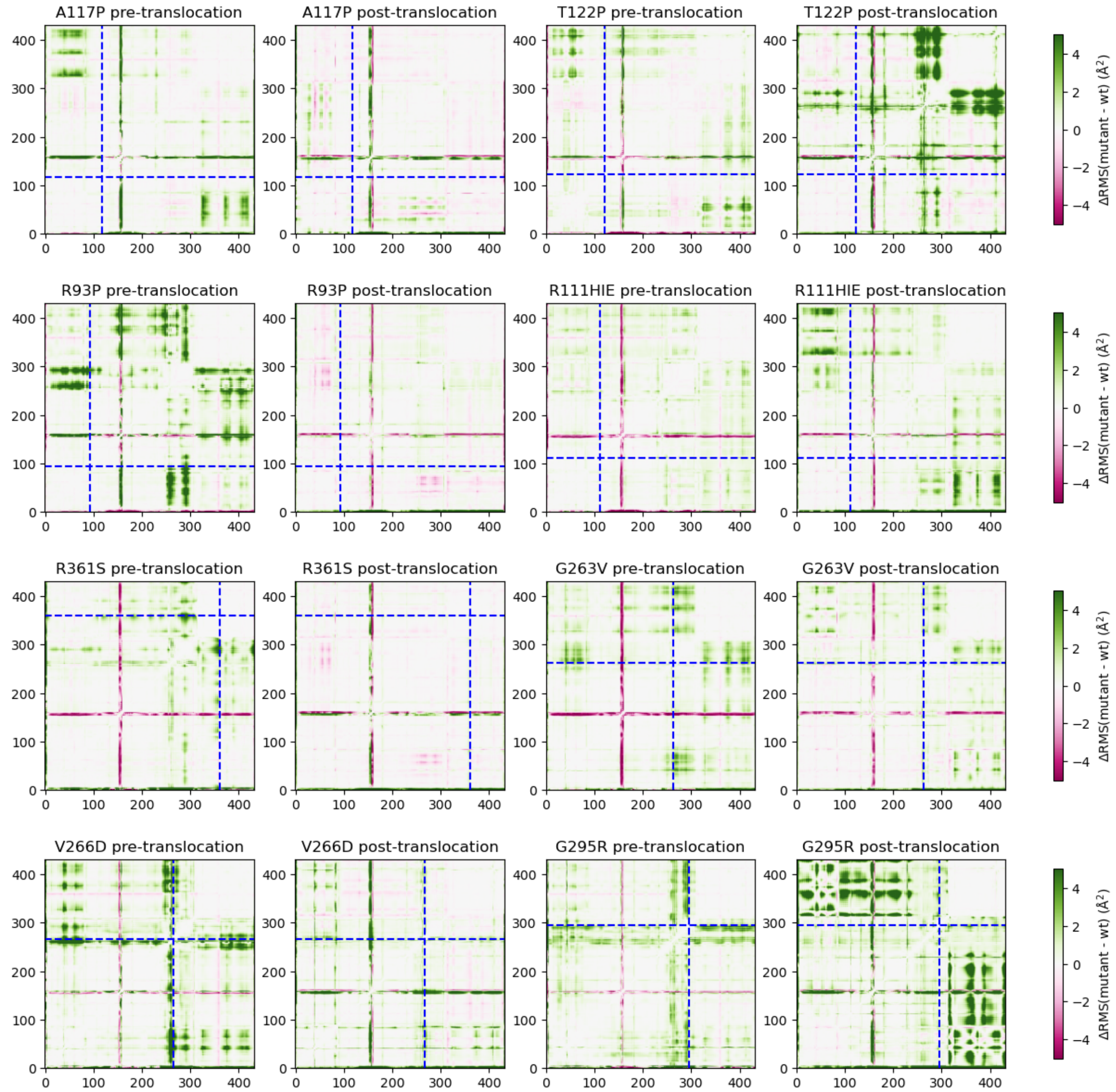

Figure S32.

Difference Cα–Cα distance fluctuation (ΔDF) matrices for eight Polη mutations in the pre- and post-translocation states.[12] Matrices report changes in inter-residue Cα distance fluctuations relative to the corresponding wild-type system, defined as ΔRMS = RMS_{mut} - RMS_{WT}, computed from MD trajectories. Values are mapped onto a diverging color scale from fuchsia (negative RMS, reduced fluctuations relative to WT) to green (positive RMS, increased fluctuations relative to WT). The position of the mutated residue is indicated by two blue dashed lines, highlighting that changes in fluctuation patterns are not necessarily localized at the mutation site but may extend to distal regions of the protein.

REFERENCES

(1) Masutani, C.; Kusumoto, R.; Yamada, A.; Dohmae, N.; Yokoi, M.; Yuasa, M.; Araki, M.; Iwai, S.; Takio, K.; Hanaoka, F. The XPV (Xeroderma Pigmentosum Variant) Gene Encodes Human DNA Polymerase η. *Nature* **1999**, *399* (6737), 700–704. https://doi.org/10.1038/21447.

(2) Broughton, B. C.; Cordonnier, A.; Kleijer, W. J.; Jaspers, N. G. J.; Fawcett, H.; Raams, A.; Garritsen, V. H.; Stary, A.; Avril, M.-F.; Boudsocq, F.; Masutani, C.; Hanaoka, F.; Fuchs, R. P.; Sarasin, A.; Lehmann, A. R. Molecular Analysis of Mutations in DNA Polymerase η in Xeroderma Pigmentosum-Variant Patients. *Proc. Natl. Acad. Sci.* **2002**, *99* (2), 815–820. https://doi.org/10.1073/pnas.022473899.

(3) Opletalova, K.; Bourillon, A.; Yang, W.; Pouvelle, C.; Armier, J.; Despras, E.; Martin, L.; Mateus, C.; Robert, C.; Kannouche, P.; Soufir, N.; Sarasin, A. Correlation of Phenotype/Genotype in a Cohort of 23 Xeroderma Pigmentosum-Variant Patients Reveals 12 New Disease-Causing POLH Mutations. *Hum. Mutat.* **2014**, *35* (1), 117–128. https://doi.org/10.1002/humu.22462.

(4) Tanioka, M.; Masaki, T.; Ono, R.; Nagano, T.; Otoshi-Honda, E.; Matsumura, Y.; Takigawa, M.; Inui, H.; Miyachi, Y.; Moriwaki, S.; Nishigori, C. Molecular Analysis of DNA Polymerase Eta Gene in Japanese Patients Diagnosed as Xeroderma Pigmentosum Variant Type. *J. Investig. Dermatol.* **2007**, *127* (7), 1745–1751. https://doi.org/10.1038/sj.jid.5700759.

(5) Miller, B. R.; McGee, T. D.; Swails, J. M.; Homeyer, N.; Gohlke, H.; Roitberg, A. E. MMPBSA.Py: An Efficient Program for End-State Free Energy Calculations. *Journal of Chemical Theory and Computation* **2012**, *8*, 3314–3321. https://doi.org/10.1021/ct300418h/asset/images/large/ct-2012-00418h_0003.jpeg.

(6) Visigalli, A.; Trizio, E.; Bonati, L.; Vidossich, P.; Parrinello, M.; De Vivo, M. Coordinated Residue Motions at the Enzyme–Substrate Interface Promote DNA Translocation in Polymerases. *J. Am. Chem. Soc.* **2025**, *147* (26), 22972–22985. https://doi.org/10.1021/jacs.5c05888.

(7) Michaud-Agrawal, N.; Denning, E. J.; Woolf, T. B.; Beckstein, O. MDAnalysis: A Toolkit for the Analysis of Molecular Dynamics Simulations. *J. Comput. Chem.* **2011**, *32* (10), 2319–2327. https://doi.org/10.1002/jcc.21787.

(8) Case, D. A.; Aktulga, H. M.; Belfon, K.; Cerutti, D. S.; Cisneros, G. A.; Cruzeiro, V. W. D.; Forouzesh, N.; Giese, T. J.; Götz, A. W.; Gohlke, H.; Izadi, S.; Kasavajhala, K.; Kaymak, M. C.; King, E.; Kurtzman, T.; Lee, T.-S.; Li, P.; Liu, J.; Luchko, T.; Luo, R.; Manathunga, M.; Machado, M. R.; Nguyen, H. M.; O'Hearn, K. A.; Onufriev, A. V.; Pan, F.; Pantano, S.; Qi, R.; Rahnamoun, A.; Risheh, A.; Schott-Verdugo, S.; Shajan, A.; Swails, J.; Wang, J.;

Wei, H.; Wu, X.; Wu, Y.; Zhang, S.; Zhao, S.; Zhu, Q.; Cheatham, T. E.; Roe, D. R.; Roitberg, A.; Simmerling, C.; York, D. M.; Nagan, M. C.; Merz, K. M. AmberTools. *J. Chem. Inf. Model.* **2023**, *63* (20), 6183–6191. https://doi.org/10.1021/acs.jcim.3c01153.

(9) Abraham, M. J.; Murtola, T.; Schulz, R.; Páll, S.; Smith, J. C.; Hess, B.; Lindahl, E. GROMACS: High Performance Molecular Simulations through Multi-Level Parallelism from Laptops to Supercomputers. *SoftwareX* **2015**, *1*, 19–25. https://doi.org/10.1016/j.softx.2015.06.001.

(10) Siemers, M.; Lazaratos, M.; Karathanou, K.; Guerra, F.; Brown, L. S.; Bondar, A.-N. Bridge: A Graph-Based Algorithm to Analyze Dynamic H-Bond Networks in Membrane Proteins. *J. Chem. Theory Comput.* **2019**, *15* (12), 6781–6798. https://doi.org/10.1021/acs.jctc.9b00697.

(11) Siemers, M.; Bondar, A.-N. Interactive Interface for Graph-Based Analyses of Dynamic H-Bond Networks: Application to Spike Protein S. *J. Chem. Inf. Model.* **2021**, *61* (6), 2998–3014. https://doi.org/10.1021/acs.jcim.1c00306.

(12) Morra, G.; Potestio, R.; Micheletti, C.; Colombo, G. Corresponding Functional Dynamics across the Hsp90 Chaperone Family: Insights from a Multiscale Analysis of MD Simulations. *PLoS Comput. Biol.* **2012**, *8* (3), e1002433. https://doi.org/10.1371/journal.pcbi.1002433.
